# Deep learning as a tool for neural data analysis: Speech classification and cross-frequency coupling in human sensorimotor cortex

**DOI:** 10.1371/journal.pcbi.1007091

**Published:** 2019-09-16

**Authors:** Jesse A. Livezey, Kristofer E. Bouchard, Edward F. Chang

**Affiliations:** 1 Biological Systems and Engineering Division, Lawrence Berkeley National Laboratory, Berkeley, California, United States of America; 2 Redwood Center for Theoretical Neuroscience, University of California, Berkeley, Berkeley, California, United States of America; 3 Helen Wills Neuroscience Institute, University of California, Berkeley, Berkeley, California, United States of America; 4 Department of Neurological Surgery and Department of Physiology, University of California, San Francisco, San Francisco, California, United States of America; 5 Center for Integrative Neuroscience, University of California, San Francisco, San Francisco, California, United States of America; 6 UCSF Epilepsy Center, University of California, San Francisco, San Francisco, California, United States of America; Stanford University, UNITED STATES

## Abstract

A fundamental challenge in neuroscience is to understand what structure in the world is represented in spatially distributed patterns of neural activity from multiple single-trial measurements. This is often accomplished by learning a simple, linear transformations between neural features and features of the sensory stimuli or motor task. While successful in some early sensory processing areas, linear mappings are unlikely to be ideal tools for elucidating nonlinear, hierarchical representations of higher-order brain areas during complex tasks, such as the production of speech by humans. Here, we apply deep networks to predict produced speech syllables from a dataset of high gamma cortical surface electric potentials recorded from human sensorimotor cortex. We find that deep networks had higher decoding prediction accuracy compared to baseline models. Having established that deep networks extract more task relevant information from neural data sets relative to linear models (i.e., higher predictive accuracy), we next sought to demonstrate their utility as a data analysis tool for neuroscience. We first show that deep network’s confusions revealed hierarchical latent structure in the neural data, which recapitulated the underlying articulatory nature of speech motor control. We next broadened the frequency features beyond high-gamma and identified a novel high-gamma-to-beta coupling during speech production. Finally, we used deep networks to compare task-relevant information in different neural frequency bands, and found that the high-gamma band contains the vast majority of information relevant for the speech prediction task, with little-to-no additional contribution from lower-frequency amplitudes. Together, these results demonstrate the utility of deep networks as a data analysis tool for basic and applied neuroscience.

## Author summary

It has been demonstrated that deep networks can achieve state of the art results on a number of classic machine learning tasks, but it is not currently clear whether deep networks can enjoy the same success in science, where not only accuracy but scientific understanding is desired. For example, the relationship between neural features and features of speech is often examined through the use of single-layer statistical models. However, single-layer models are unlikely to be able to describe the complex representations in higher-order brain areas during speech production. In this study, we show that deep networks achieve state of the art accuracy when classifying speech syllables from the amplitude of cortical surface electrical potentials. Furthermore, deep networks reveal an articulatory speech hierarchy consistent with previously studies which used hand-designed features. We also report a novel positive coupling between the beta and high-gamma bands during speech production in “active” cortical areas. However, using deep networks we show that, compared to lower frequency bands, the high gamma amplitude is by far the most informative signal for classifying speech.

## Introduction

A central goal of neuroscience is to understand what and how information about the external world (e.g., sensory stimuli or behaviors) is present in spatially distributed, dynamic patterns of brain activity. At the same time, neuroscience has been on an inexorable march away from the periphery (e.g., the retina, spinal cord), seeking to understand higher-order brain function (such as speech). The methods used by neuroscientists are typically based on simple linear transformations, which have been successful predictors in early processing stages of the nervous system for simple tasks [1–3]. However, linear methods are limited in their ability to represent complex, hierarchical, nonlinear relationships [4], which are likely present in the neural activity of higher-order brain areas. This linear restriction may not only limit the predictive accuracy of models but may also limit our ability to uncover structure in neural datasets.

Multilayer deep networks can combine features in nonlinear ways when making predictions. This gives them more expressive power in terms of the types of mappings they can learn at the cost of more model hyperparameters, more model parameters to train, and more difficult training dynamics [5]. Together with the recent success of deep learning in a number of fields including computer vision, text translation, and speech recognition [6–8], the ability of deep networks to learn nonlinear function from data motivates their use for understanding neural signals. The success of deep learning in classic machine learning tasks has spurred a growth of applications into new scientific fields. Other nonlinear methods such as random trees/forests can also be used on nonlinear neural data but often require more feature selection/reduction and are not typically used on data with thousands or tens of thousands of features [9]. Deep networks have recently been applied as classifiers for diverse types of physiological data including electromyographic (EMG), electroencephalographic (EEG), and spike rate signals [10–13], on stimulus reconstruction in sensory regions using electrocorticography (ECoG) [14], as models for sensory and motor systems [15–19]. Compared to datasets used in traditional machine learning, neuroscientif datasets tend to be very small. As such, models in neuroscience tend to be smaller (fewer layers, units per layers) and in this sense more similar to neural networks from previous decades. However, modern deep learning techniques such as ReLUs, Dropout, and optimization algorithms like Nesterov momentum are crucial to train them to high held-out performance. While these studies have demonstrated the superior performance of deep networks as black-box predictors, the utilization of deep networks to gain understanding into brain computations is rare. Whether deep networks can be used to elucidate the latent structure of scientific and neuroscientific datasets is still an open question.

Vocal articulation is a complex task requiring the coordinated orchestration of several parts of the vocal tract (e.g., the larynx, tongue, jaw, and lips). To study the neural basis of speech requires monitoring cortical activity at high spatio-temporal resolution (on the order of tens of milliseconds) over large areas of sensorimotor cortex (∼1300mm^2^) [20]. Electrocorticography (ECoG) is an ideal method to achieve the simultaneous high-resolution and broad coverage requirements in humans. Using such recordings, there has been a surge of recent efforts to understand the cortical basis of speech production [20–25]. For example, analyzing mean activity, Bouchard et. al. [20] demonstrated, much in the spirit of Penfield’s earlier work [26], that the ventral sensorimotor cortex (vSMC) has a spatial map of articulator representations (i.e. lips, jaw, tongue, and larynx) that are engaged during speech production. Additionally, it was found that spatial patterns of activity across the vSMC network (extracted from trial average activity with principal components analysis at specific time points) organized phonemes along phonetic features emphasizing the articulatory requirements of production.

Understanding how well cortical surface electrical potentials (CSEPs) capture the underlying neural processing involved in speech production is important for revealing the neural basis of speech and improving speech decoding for brain-computer interfaces [27, 28]. Previous studies have used CSEPs and linear or single layer models to predict speech categories [23, 29–32], or continuous aspects of speech production (e.g., vowel acoustics or vocal tract configurations) [22, 25], with some success. However, given the challenge of collecting large number of samples across diverse speech categories, it is not clear that we should expect high performance from deep networks for speech classification. Exploring the use of deep networks to maximally extract information for speech prediction is not only important for brain machine interfaces which restore communication capabilities to people who are “locked-in”, but also for identifying cortical computations which are the underlying basis for speech production.

In general, understanding information content across neural signals, such as different frequency components of CSEPs, is an area of ongoing research [33–37]. A number of studies have found relationships between different frequency components in the brains electrical potentials. These can take the form of phase and amplitude structure of beta (*β*) waves [38, 39] or correlations between lower frequency oscillations and spiking activity or high gamma (H*γ*) activity [37, 40]. One observation is that *β* band (14-30Hz) amplitude and coherence [33, 41] often decreases during behavior, when the state is changing [42]. This has lead to the interpretation that *β* may be serving a “maintenance of state” function. However, often these effects are not differentiated between functional areas that are active versus inactive during behavior. Indeed, in other contexts, aggregation has been shown to mask structure in neural signals [43]. The somatotopic organization of speech articulator control in human vSMC, and the differential engagement of these articulators by different speech sounds, potentially provides the opportunity to disentangle these issues. Furthermore, classifying behaviors, such as speech, from CSEPs can be used as a proxy for information content in a signal, obfuscating the interpretation of the results. However, this is often done using linear methods, which may not be able to take full advantage of the information in a signal. Since deep networks are able to maximize classification performance, they are an ideal candidate for comparing information content across neural signals.

In this work, we investigated deep networks as a data analytics framework for systems neuroscience, with a specific focus on the uniquely human capacity to produce spoken language. First, we show that deep networks achieve superior classification accuracy compared to linear models, with increased gains for increasing task complexity. We then “opened the black box” and used the deep network confusions to reveal the latent structure learned from single trials, which revealed a rich, hierarchical organization of linguistic features. Since deep networks classified speech production from H*γ* activity with higher accuracy that other methods, they are also candidates for determining the relative information content across neural signals. We explored the cross-frequency amplitude-amplitude structure in the CSEPs and discovered a novel signature of motor coordination in *β*-H*γ* coupling. Using deep networks, we then show that although there is information relevant to speech production in the lower frequency bands, it is small compared to H*γ*. Critically, the lower frequency bands do not add significant additional information about speech production about and beyond H*γ*. Furthermore, the correlations are not tightly related to overall information content and improvements in accuracy. Together, these results demonstrate the utilization of deep networks not only as an optimal black-box predictor, but as a powerful data analytics tool to reveal the latent structure of neural representations, and understanding the information content of different neural signals.

## Materials and methods

### Experimental data

The experimental protocol, collection, and processing of the data examined here have been described in detail previously [20–22]. The experimental protocol was approved by the Human Research Protection Program at the University of California, San Francisco. Briefly, four native English speaking human subjects underwent chronic implantation of a subdural electrocortigraphic (ECoG) array over the left hemisphere as part of their clinical treatment of epilepsy. The subjects gave their written informed consent before the day of surgery. The subjects read aloud consonant-vowel (CV) syllables composed of 19 consonants followed by one of three vowels (/a/, /i/ or /u/), for a total of 57 potential consonant-vowel syllables. Subjects did not produce each CV in an equal number of trials or produce all possible CVs. Across subjects, the number of repetitions per CV varied from 10 to 105, and the total number of usable trials per subject was S1: 2572, S2: 1563, S3: 5207, and S4: 1422. CVs for which there was not enough data to do cross-validation (fewer than 10 examples) were excluded per-subject.

### Signal processing

Cortical surface electrical potentials (CSEPs) were recorded directly from the cortical surface with a high-density (4mm pitch), 256-channel ECoG array and a multi-channel amplifier optically connected to a digital signal processor (Tucker-Davis Technologies [TDT], Alachua, FL). The time series from each channel was visually and quantitatively inspected for artifacts or excessive noise (typically 60 Hz line noise). These channels were excluded from all subsequent analysis and the raw CSEP signal from the remaining channels were downsampled to 400 Hz in the frequency domain and then common-average referenced and used for spectro-temporal analysis. For each useable channel, the time-varying analytic amplitude was extracted from 40 frequency domain, bandpass filters (Gaussian filters, logarithmically increasing center frequencies and semi-logarithmically increasing band-widths, equivalent to a frequency domain Morlet wavelet). The amplitude for each filter band was z-scored to a baseline window defined as a period of time in which the subject was silent, the room was silent, and the subject was resting. Finally, the amplitudes were downsampled to 200 Hz.

For each of the bands defined as: theta [4-7 Hz], alpha [8-14 Hz], beta [15-29 Hz], gamma [30-59 Hz], and high gamma [70-150 Hz], individual bands from the 40 Gaussian bandpassed amplitudes were grouped and averaged according to their center frequencies. The lower frequency features are all highly oversampled at the H*γ* rate of 200 Hz. To make comparisons across frequency bands more interpretable, control for potential overfitting from training on oversampled signals, and to reduce the computational complexity of training deep networks with concatenated input features, we downsampled each of the lower frequency bands in time so that the center frequency-to-sampling rate ratio was constant (ratio = 112.5/200) for each band. Given limited data, deep networks are tasked with deciding whether a change across input features is relevant or irrelevant for prediction. The lower frequency bands are highly oversampled at 200 Hz, however, the higher frequencies will not have exactly zero amplitude do to numerical noise even though these are irrelevant signals. Downsampling the bands to a fixed ratio makes comparing CV decoding accuracy across frequency bands more interpretable.

Based on previous results [20–22], we focused on the electrodes in the ventral sensorimotor cortex (vSMC). The activity for each of the examples in our data set was aligned to the acoustic onset of the consonant-to-vowel transition. For each example, a window 0.5 seconds preceding and 0.79 seconds following the acoustic onset of the consonant-to-vowel transition was extracted. The mean of the first and last ∼ 4% time samples was subtracted from the data per electrode and trial (another form of amplitude normalization that is very local in time). This defined the z-scored amplitude that is used for subsequent analyses.

### Deep networks

Supervised classification models often find their model parameters, $\hat{\Theta }$, which minimize the negative log-likelihood of the training data and labels, {*x*^(*i*)^, *y*^(*i*)^}, under a model which gives the conditional probability of the labels given the input data

$$\begin{array}{c} \hat{\Theta }=\underset{\Theta }{\text{arg}\;\text{min}}\;-\text{log}\;P\left(Y|X;\Theta \right),\;\left\{x^{\left(i\right)},y^{\left(i\right)}\right\}. \end{array}$$ (1)

Deep networks typically parametrize this conditional probability with a sequence of linear-nonlinear operations. Each layer in a fully-connected network consists of an affine transform followed by a nonlinearity:

$$\begin{array}{c} \begin{array}{cc} h^{1} & =f(w^{1}\cdot x+b^{1}),\\ h^{i} & =f(w^{i}\cdot h^{i-1}+b^{i}),\;\text{with}\\ \Theta & =\{w_{1},\ldots ,w_{n},b_{1},\ldots ,b_{n}\} \end{array} \end{array}$$ (2)

where *x* is a batch of input vectors, *w*^*i*^ and *b*^*i*^ are trainable parameters (weights and biases, respectively) for the *i*th layer, *h*^*i*^ is the *i*th hidden representation, and *f*(⋅) is a nonlinearity which can be chosen during hyperparameter selection. Single layer classification methods, such as multinomial logistic regression, are a special case of deep networks with no hidden representations and their corresponding hyperparameters.

For the fully-connected deep networks used here, the CSEP features were rasterized into a large feature vector per-trial in a window around CV production. These feature vectors are the input into the first layer of the fully connected network. The feature dimensionality is the number of electrodes by 258 time points which corresponds to Subject 1: 22,188, Subject 2: 20,124, Subject 3: 21,414, and Subject 4: 25,542 features. The final layer non-linearity is chosen to be the softmax function:

$$\begin{array}{c} P\left({\hat{y}}_{i}\right)=\text{softmax}\left(h_{i}\right)=\frac{\text{exp}(h_{i})}{\sum_{j}\text{exp}\left(h_{j}\right)} \end{array}$$ (3)

where *h*_*i*_ is the *i*th element of the hidden representation. This nonlinearity transforms a vector of real numbers into a vector which represents a one-draw multinomial distribution. It is the negative log-likelihood of this distribution over the training data which is minimized during training.

To train and evaluate the networks, the trials were organized into 10 groupings (folds) with mutually exclusive validation and test sets and 80-10-10% splits (training, validation, testing). Since some classes may have as few as 10 examples, it was important to split each class proportionally so that all classes were equally distributed. Training terminated when the validation accuracy did not improve for 10 epochs and typically lasted about 25 epochs. Theano, Pylearn2, and Scikit-learn [44–46] were used to train all deep and linear models.

As baseline models, we used multinomial logistic regression. Logistic regression required no additional dimensionality reduction and had the highest classification accuracy compared to other linear classifiers, i.e. linear support vector machines and linear discriminant analysis on the H*γ* features (10.4 ± 6.7% and 16.0 ± 10.0% respectively compared to 28.0 ± 12.9% for logistic regression). Additionally, the conditional class distribution used in logistic regression (multinomial) is the same as the one used for deep networks, which facilitated comparison of confusions.

#### Hyperparameter search

Deep networks have a number of hyperparameters that govern network architecture and optimization such as the number of layers, the layer nonlinearity, and the optimization parameters. The full list of hyperparameters and their ranges is listed in S1 Table.

For all results that are based on training networks, 400 models were trained with hyperparameters selected with random search [47]. For each set of hyperparameters, 10 copies of the network were trained on the respective 10 folds as described in Deep networks, for a total of 4000 networks per subject per task. For each task, optimal hyperparameters were selected by choosing the model with the best mean validation classification accuracy across 10 folds. Since our datasets were relatively small for training deep networks, we regularized the models in three ways: dropout, weight decay, and filter norm-clipping in all layers of the model. The dropout rate, activation-rescaling factor, max filter norm, and weight decay coefficient were all optimized hyperparameters. The optimal values for the hyperparameters were selected independently for each family of models in the paper, i.e. independently for each subject, model type (logistic or deep), input data type (frequency bands), and amount (data scaling experiment). The search space for hyperparameters was shared across all models, however, for the logistic regression models, the number of hidden layers was set to zero and no other hidden layer hyperparameters were used. The optimal hyperparameters for each model and links to trained model files and Docker images for running preprocessing and deep network training are available in S1 Appendix.

#### Classification tasks

Each subject produced a subset of the 57 CV and the classification methods were trained to predict the subset. Each CV can also be classified as containing 1 of 19 consonants or 1 of 3 vowels. Similarly, a subset of the constants can be grouped into 1 of 3 vocal tract constriction location categories or 1 of 3 vocal tract constriction degree categories. The CV predictions were then tabulated within these restricted labelings in order to calculate the accuracy for consonant, vowel, constriction location, and constriction degree accuracies.

As there are drastically different numbers of classes between the different tasks (between 3 and 57), as well as subtle differences between subjects, classification accuracies and changes in accuracies are all normalized to chance. In each case, chance accuracy is estimated by assuming that test set predictions are drawn randomly from the training set distribution. This process was averaged across 100 random resamplings per fold, training fraction, subject, etc. Estimating chance accuracy by training models on data with shuffled labels was not possible for consonant constriction location and degree tasks since not all CVs were part of the restricted task and occasionally networks would predict 0 trials within the restricted task which would give undefined chance accuracy.

On the CV task, we compared performance scaling of different models by training on different fractions of the training set. For each fraction of the data, each class was subsampled individually to ensure all classes were present in the training set. The aggregate slopes were calculated with ordinary least-squares regression. The validation and test sets were not subsampled. Hyperparameters were chosen independently for each fraction of the training data.

#### Information content in neural signals

For a given experimentally defined behavior, such as CV speech production, the information about the task is presumably present in the activity of the brain, which we coarsely measure with different frequency components of the recorded CSEPs. The information about the task in the measurements can be formalized by the mutual information between the task variable *Y* and the neural measurement variable *X* [48]

$$\begin{array}{c} I\left(Y;X\right)=\sum_{y,x}P\left(y,x\right)\;{\text{log}}_{2}(\frac{P(y,x)}{P(y)P(x)}). \end{array}$$ (4)

It is not possible to calculate this quantity directly because we do not know the joint distribution of neural measurements and speech tokens, *P*(*X*, *Y*) and cannot easily approximate it due to the small number of samples (∼ 10^3^) compared to the dimensionality of each measurement (∼ 10^4^). However, we can classify the behavior from the neural data using statistical-machine learning methods, i.e. deep learning. For a supervised classification task, machine learning methods typically generate conditional probabilities $P(\hat{Y}|X)$. Since we know the ground-truth behavior for each measurement, we can use the classifier to compute the mutual information between the behavior state, *Y*, and the predicted state, $\hat{Y}$

$$\begin{array}{c} I\left(\hat{Y};Y\right)=\sum_{y,\hat{y}}P\left(\hat{y}|y\right)P\left(y\right)\;{\text{log}}_{2}(\frac{P\left(\hat{y}|y\right)P\left(y\right)}{P\left(y\right)P\left(\hat{y}\right)}). \end{array}$$ (5)

The data processing inequality tells us that this quantity is a lower bound to *I*(*Y*;*X*).

Given this lower bound, if everything else is held constant, the classification method with highest accuracy will lead the tightest estimate of the mutual information between the task and neural data, *I*(*Y*; *X*), which is a quantity that is relevant for future experimental hardware, methods, and data preprocessing development.

This quantity is closely related to a second measure of classifier performance, the Channel Capacity (CC). To compare our results with previous speech classification studies, we report estimated CC, which is measured in bits per symbol, in addition to classification statistics. CC is a unified way of calculating the effectiveness of different speech classifiers, which can have differing numbers of classes and modalities. The channel capacity, *CC*, between the ground truth class, *Y*, and predicted class, $\hat{Y}$, is defined as:

$$\begin{array}{c} CC=\underset{P(Y)}{\text{supremum}}\;I\left(\hat{Y};Y\right)=\underset{P(Y)}{\text{supremum}}\sum_{{\hat{y}}_{i},y_{j}}P\left({\hat{y}}_{i}|y_{j}\right)P\left(y_{j}\right)\;{\text{log}}_{2}(\frac{P({\hat{y}}_{i}|y_{j})}{P({\hat{y}}_{i})}). \end{array}$$ (6)

For previous work, we must approximate the channel capacity since we do not have access to the details of the classification performance, $P(\hat{Y}|Y)$. Wolpaw et. al. [49] suggest an approximation that assumes all classes have the same accuracy as the mean accuracy and all errors are distributed equally (note that this second assumption is generally not true in speech, i.e. Fig 4C, also noted in [23]). To make a fair comparison, we compute this approximate value for our results in addition to the exact value. For our data, we find that the approximation underestimates the true channel capacity for the CV and consonant task. The Information Transfer Rate (ITR) is also commonly reported, which is the channel capacity divided by the symbol duration in time. Since we are considering fixed length measurements (1.3 s), we report channel capacity rather than ITR.

### Structure of deep network predictions

Neuroscientists commonly study the model/confusions of linear analysis methods to gain insight into the structure of neural data. Deep networks can learn high dimensional, nonlinear features from data. Here, these features are learned by training the networks to perform classification, i.e. maximize $P({\hat{Y}}_{i}|X_{i};\Theta )$ where the subscript *i* indicates true class membership. It has been shown that these features contain more information than the thresholded multinomial classification prediction [50, 51]. The off-diagonal values: $P({\hat{Y}}_{i}|X_{j};\Theta )$, *i* ≠ *j*, in this learned distribution represent prediction uncertainty for a given CSEP measurement. Uncertainty is learned during the training process and larger pairwise uncertainty between class labels means that the model has a harder time distinguishing those classes. Since the uncertainty (similarity) is not encoded in the supervised labels, this means that the neural data for those class labels is more similar.

To gain insight into the nature of the vSMC neural activity, we analyzed the structure of deep network predictions. The mean network prediction probabilities on the test set are used as features for each CV. A dendrogram was computed from the hierarchical clustering (Ward’s method) of these features. To aid visualization of these results, a threshold in the cluster distance was chosen by visual inspection of when the number of clusters as a function of distance rapidly increased, and the linguistic features were labeled by hand. The CV order from this clustering was used to order the features in the soft-confusion matrix and accuracy per CV. The soft confusion matrix shows mean network prediction probabilities on the test set rather than the aggregated thresholded predictions often shown in confusion matrices.

To compare the articulatory features and the deep network features quantitatively across subjects, pairwise distances between CVs were computed in both the articulatory and deep network spaces (see S1 Fig for articulatory features). These pairwise distances were then correlated per for each CV and subject and articulatory grouping.

### Cross-band amplitude-amplitude correlations

To examine the relationship between the amplitudes of different frequency components of recorded CSEPs, we first performed a correlation analysis. For this analysis, the data was trial-averaged per CV then organized into a data-tensor, *D*_CV,frequency,electrode,time_. The frequency bands were then either used individually or aggregated into canonical frequency components, such as H*γ*

$$\begin{array}{c} \bar{D}{\left(\text{H}\gamma \right)}_{\text{CV},\text{electrode},\text{time}}={\left\langle {\bar{D}}_{\text{CV},\text{frequency},\text{electrode},\text{time}}\right\rangle }_{\text{frequency}\in \text{H}\gamma }. \end{array}$$ (7)

$\bar{D}{\left(\text{H}\gamma \right)}_{\text{CV},\text{electrode},\text{time}}$ was correlated across time at 0 ms lag with each of the 40 Gaussian bandpassed amplitudes averaged across CVs and electrodes. The correlation between $\bar{D}{\left(\text{H}\gamma \right)}_{\text{CV},\text{electrode},\text{time}}$ and $\bar{D}{\left(\beta \right)}_{\text{CV},\text{electrode},\text{time}}$ was computed and histogrammed across CVs and electrodes. The average H*γ* power was averaged in a window 70 ms before and 140 ms after the CV acoustic transition and histogrammed across CVs and electrodes. This window was chosen as it is the most active and informative time period for consonants and vowels.

### Resolved cross-band amplitude-amplitude correlation

Since the ECoG grid covers a large functional area of vSMC and the CV task differentially engages articulators for different consonant and vowels, the correlations can be computed independently for “active” versus “inactive” electrodes for each CV (averaged across trials). To define active and inactive electrode groups for a band, B and H*γ*, first, the B-Hγ amplitude-amplitude correlation, C(B,Hγ) and average H*γ* amplitude, *A*(H*γ*), with positive average amplitude (greater than baseline) are used to fit one linear model with ordinary least-squares regression

$$\begin{array}{c} \hat{m},\hat{b}=\underset{m,b}{\text{arg}\;\text{max}}\sum_{ij}{\left(C_{ij}\left(B,\text{H}\gamma \right)-\left(mA_{ij}\left(\text{H}\gamma \right)+b\right)\right)}^{2} \end{array}$$ (8)

for all electrodes, *i*, and CVs, *j*. The electrodes were then divided into “active” and “inactive” per CV by thresholding the average H*γ* activity where the linear fit predicted 0 correlation.

$$\begin{array}{c} A_{\text{thresh}}\left(\text{H}\gamma \right)=-\frac{\hat{b}}{\hat{m}}. \end{array}$$ (9)

Electrodes with average H*γ* activity above threshold were active, and those with lower average H*γ* activity were inactive. The active and inactive electrodes per CV were separated and $\bar{D}{\left(\text{H}\gamma \right)}_{\text{CV},\text{electrode},\text{time}}$ was correlated across time at 0 ms lag with each of the 40 Gaussian bandpassed amplitudes averaged across CVs and electrodes independently for the active and inactive electrodes and for each subject.

### Classification from other frequency bands

An extended sets of lower frequency features per trial were used in addition to the H*γ* features for each of the theta, alpha, low beta, high beta, and gamma bands. The lower frequency amplitudes are highly oversampled at 200 Hz (the H*γ* sampling frequency), and overfitting due to this mismatch will confound the interpretations of signal content. To minimize overfitting, the lower frequency amplitudes were downsampled as described in the Signal processing subsection. For each frequency band, fully-connected deep networks were trained first on the individual bands’s features and then with the band’s features concatenated with the H*γ* features. Deep network training was done in the same manner at the networks trained solely on H*γ* features. The resulting classification accuracies were then compared with the baseline H*γ* classification accuracy and then with the band’s features concatenated with the H*γ* features.

## Results

**Fig 1:**
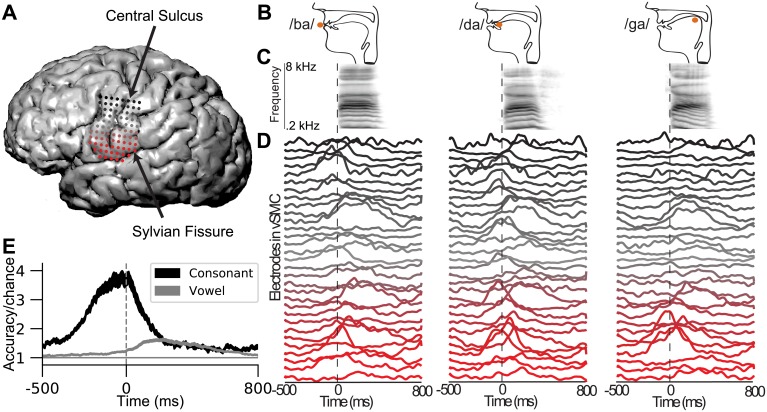
Human ECoG recordings from ventral sensorimotor cortex (vSMC) during speech production. **A** Electrodes overlaid on vSMC. Electrodes are colored red-to-black with increasing distance from the Sylvian Fissure. **B-D** Task and data summary for three different consonant-vowel (CV) utterances. **B** Vocal tract configuration and point of constriction (orange dot) during the consonant for the production of /ba/ (lips), /da/ (coronal tongue), and /ga/ (dorsal tongue). **C)** The audio spectrogram aligned to the consonant-to-vowel acoustic transition (dashed line). **D** Mean across trials of the H*γ* amplitude from a subset of electrodes in vSMC aligned to CV transition. Traces are colored red-to-black with increasing distance from the Sylvian Fissure as in **A**. The syllables /ba/, /da/, and /ga/ are generated by overlapping yet distinct spatio-temporal patterns of activity across vSMC. **E** Logistic regression accuracy for consonants and vowels plotted against time aligned to the CV transition averaged across subjects and folds. Black and grey traces are average (± s.e.m., *n* = 40) accuracies for consonants (18–19 classes) and vowels (3 classes) respectively.

A subset of the electrodes of the ECoG grid overlaid on the vSMC of Subject 1 is shown in Fig 1A. Cortical electric surface potentials (CSEPs) were recorded from the left hemisphere of 4 subjects during the production of a set of consonant-vowel syllables which engage different section of the vocal tract, as shown in Fig 1B, to produce acoustics which are shown in Fig 1C. The trial-averaged z-scored high gamma (H*γ*) amplitude recorded during the production of the syllables from Fig 1B show spatially and temporally distributed patterns of activity (Fig 1D). Here we see that cortical surface electrical potentials recorded from vSMC during the production of CVs consists of multiple spatially and temporally overlapping patterns.

Spatiotemporal patterns of activity represent information about the produced syllables [20]. This is shown by training multinomial logistic regression models independently at each time point using all electrodes in vSMC (Fig 1E). Across subjects, the consonant classification accuracy rises from chance approximately 250 ms before the consonant-vowel acoustic transition at 0 ms, which precedes the acoustic production of the consonants, indicating the motor nature of the recordings. Consonant classification accuracy remains above chance for approximately 200 ms into vowel acoustics production. Vowel classification accuracy rises just before the transition to vowel acoustics production and remains above chance for approximately 500 ms. These results show that the consonant and vowel identity is encoded in the H*γ* amplitude in partially-overlapping temporal segments.

### Deep learning for speech classification

#### Deep networks outperform standard methods for consonant-vowel classification from high gamma amplitude

It has been shown that CSEPs contain information about motor control [20, 22, 23, 29, 31, 52] and variability [21]. Regressing CSEP time-frequency features onto behavioral features with linear methods has been used to elucidate the information content. Linear decoders can put a lower bound on the behaviorally relevant information in a measurement, but the restriction to linear mappings may limit the amount of information they are able to extract from the neural signal.

**Fig 2:**
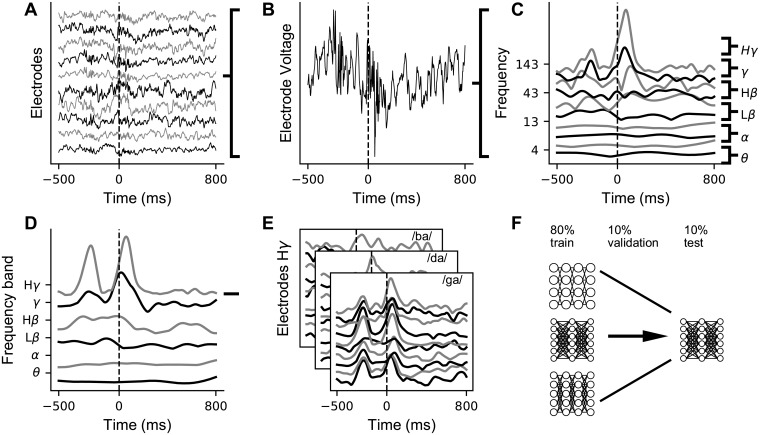
Data processing and deep network training pipeline for ECoG data. **A** Cortical surface electrical potentials plotted against time for a subset of the vSMC electrodes segmented to the CV production window. Electrodes have an arbitrary vertical offset for visualization. **B** Voltage for one electrode. **C** The z-scored analytic amplitude is shown for a subset of the 40 frequency ranges used in the Hilbert Transform as a function of time. **D** The 40 ranges used in the Hilbert Transform are grouped and averaged according to whether their center frequency is part of each traditional neuroscience band. **E** For a particular analysis, a subset of the bands are chosen as features, and this process was repeated for each trial (sub-pane) and electrode (trace within each sub-pane) in vSMC. Each data sample consists of one trial’s H*γ* activity for all electrodes in vSMC. **F** Data were partitioned 10 times into training, validation, and testing subsets (80%, 10%, and 10% respectively) with independent testing subsets. We trained models that varied in a large hyper-parameter space, including network architecture and optimization parameters, symbolized by the 3 networks on the left with differing numbers of units and layers. The optimal model (right) is chosen based on the validation accuracy and results are reported on the test set.

Deep networks can learn more complex, nonlinear mappings, which can potentially extract more information from a neural signal. Thus, they may be able to put a tighter lower bound on the information relevant for speech classification contained in CSEP features. To test this, fully connected deep networks and baseline multinomial logistic regression models were trained on z-scored *Hγ* amplitude from all electrodes in vSMC and time points in a window around CV production. Fig 2 shows how the raw CSEP measurements are preprocessed into time-frequency features across behavioral trials, selected and grouped into datasets, and are used in the deep network hyperparameter cross-validation loop. The networks with the highest validation accuracy, averaged across 10 folds, were selected and their results on a held-out test set are reported.

**Fig 3:**
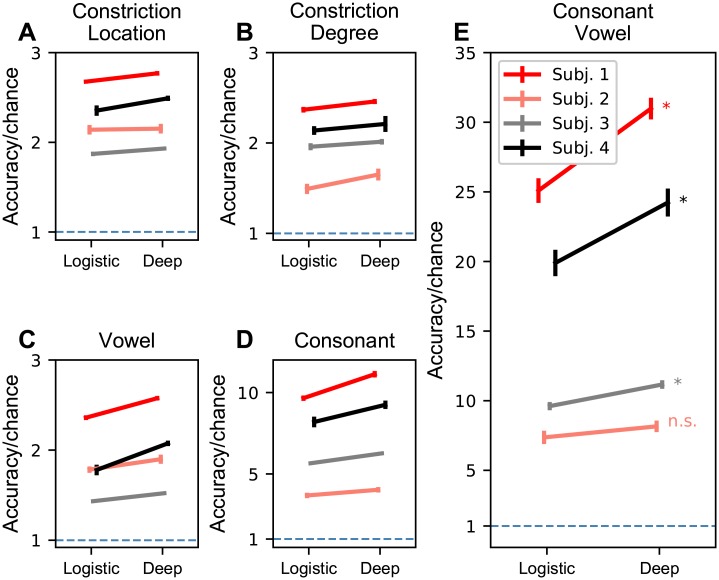
Classification accuracy of logistic regression versus deep networks for different classification tasks. For **A**-**E**, accuracies (± s.e.m., n = 10) are normalized to chance (chance = 1, dashed blue line) independently for each subject and task. Points on the left are multinomial logistic regression accuracy and are connected to the points on the right which are deep network accuracies for each subject. Subject accuracies have been left-right jittered to prevent visual overlap and demarcated with color (legend in **E**). **A-D** Classification accuracy when CV predictions are restricted to consonant constriction location (**A**), consonant constriction degree (**B**), vowel (**C**), or consonant (**D**) classification tasks. **E** Classification of entire consonant-vowel syllables from H*γ* amplitude features. *p < 0.05, WSRT, Bonferroni corrected with *n* = 4. n.s., not significant. Significance was tested between deep network and logistic regression accuracies.

Behaviorally, speech is organized across multiple levels. Even within the simple CV task examined here, there are multiple levels of attributes that can be associated with each CV syllable. The simplest description of the CVs correspond to the consonant constriction location, consonant constriction degree, or vowel labels (3-way tasks). Fig 3A–3C shows the accuracy in these cases respectively. For these tasks, subjects with baseline accuracy close to chance see little-to-no improvement and subjects with larger improvements are limited by the low complexity of the 3-way classification task. In order to partially normalize task complexity across tasks with very different numbers of outcome possibilities (and hence different chance levels), accuracy/chance is shown in Fig 3. This normalization highlights performance on tasks with higher complexity, e.g., CV classification, which would otherwise have lower accuracy than simpler tasks, e.g., vowel classification. S2 Fig shows this same data plotted as raw accuracy. An intermediate level of complexity is the consonant label (18 or 19-way, Fig 3D). The highest deep network accuracy for a single subject on the consonant task is for Subject 1 which is 59.0 ± 2.2% (11.1 times chance, 5.3%) and 51.2 ± 1.8% (9.7 times chance, 5.3%) for logistic regression and deep networks respectively, which is a 15.3% improvement. Mean consonant classification accuracy across subjects (19 way) with deep networks is 41.2 ± 14.3%. For logistic regression, it is 36.5 ± 12.3%.

Finally, the most complex task is CV classification which has between 54 and 57 classes across subjects. The highest deep network accuracy for a single subject on the consonant vowel task is for Subject 1 which is 55.1 ± 2.3% (31.0 times chance, 1.8%) and 44.6 ± 3.2% (25.1 times chance, 1.8%) for logistic regression and deep networks respectively, which is a 24.0% improvement (Fig 3E). Mean consonant vowel classification accuracy across subjects (54-57 way) with deep networks is 33.7 ± 16.4%. For logistic regression, it is 28.0 ± 12.9%. Per subject improvements (change in accuracy normalized to chance) for Subjects 1 through 4 are 5.9x (*p* < 0.05), 0.8x (n.s.), 1.6x (*p* < 0.05), and 4.3x (*p* < 0.05). For each subject, a Wilcoxon Signed-Rank Test (WSRT) was performed and the resulting p value was Bonferroni corrected (*n* = 4). For the 3 significant results, the p-value was at the floor for a WSRT with *n* = 10 samples and no equal differences.

The results described above contain many potential sources of variation. To test the significance of these variations, we use an ANOVA with subject, model type (deep network versus logistic regression), task complexity (CV versus consonant versus vowel, location, degree), and model-task complexity interaction as categorical groupings. This model is significant (f-statistic: 177.0, *p* < 1 × 10^−10^) and all coefficients were significant at *p* < 0.001 except for the deep network-consonant interaction which was significant at *p* < 0.05 with Subject 1, CV task, and logistic regression categories as the reference treatment (see S2 Table for details). This shows that deep networks are able to provide better estimate of information contained in the H*γ* amplitude as compared to linear methods.

The number of speech tokens, duration of a task, and recording modality often differ from study to study [49]. This means that quantifying the quality of speech classification from neural signals using accuracy or accuracy normalized to chance can be misleading. The Information Transfer Rate (ITR, bits per second) is a quantity that combines both accuracy and speech in a single quantity [49]. Since we are comparing fixed length syllables, this is equivalent to calculating the number of bits per syllable which can be calculated with the Channel Capacity (CC, Eq 6). The ITR can be calculated by diving the CC by the syllable duration. A summary of the accuracy results along with channel capacity estimates are summarized in Table 1 and compared against the results of Mugler et al. [23] which has a similar task and used linear discriminant analysis (LDA) as the classifier. Additional classification metrics are reported in S3 Table. Deep networks achieve state of the art classification accuracy and have the highest CC, and therefore ITR, on the full CV task. The state of the art accuracy and ITR are important quantities for brain-computer interfaces, which often limit communication rates in clinical applications.

**Table 1:** Classification and channel capacity results.

Model	Accuracy	Acc./Chance	CC (Bits/Syllable)
**Deep network, 57 CV, single subj**.	**55.1 ± 2.3%**	**31.0x**	**2.25 (3.92 exact)**
Deep network, 57 CV, subj. average	33.7 ± 16.4%	18.6x	1.15 (2.94 exact)
Logistic Regression, 57 CV, single subj.	44.6 ± 3.2%	25.1x	1.63 (3.49 exact)
Logistic Regression, 57 CV, subj. average	28.0 ± 12.9%	15.5x	0.86 (2.71 exact)
**Deep network, 19 cons., single subj**.	**59.0 ± 2.2%**	**11.1x**	**1.6 (2.42 exact)**
Deep network, 19 cons., subj. average	41.2 ± 14.3%	7.7x	0.91 (1.63 exact)
Logistic Regression, 19 cons., single subj.	51.2 ± 1.8%	9.7x	1.25 (2.04 exact)
Logistic Regression, 19 cons., subj. average	36.5 ± 12.3%	6.8x	0.72 (1.41 exact)
LDA [23], 24 cons., single subj.	36.1%	4.9x	0.75
LDA [23], 24 cons., subj. average	20.4 ± 9.8%	2.8x	0.25
**Deep network, 3 vowels, single subj**.	**85.9 ± 2.3%**	**2.6x**	**1.19 (0.87 exact)**
Deep network, 3 vowels, subj. average	67.3 ± 13.1%	2.0x	0.63 (0.45 exact)
Logistic Regression, 3 vowels, single subj.	78.7 ± 2.6%	2.4x	0.91 (0.64 exact)
Logistic Regression, 3 vowels, subj. average	61.3 ± 11.8%	1.8x	0.46 (0.32 exact)
LDA [23], 15 vowels, single subj.	23.9%	1.9x	0.22
LDA [23], 15 vowels, subj. average	19.2 ± 3.7%	1.5x	0.12

How the accuracy and precision of data analysis results scale with dataset size is an important metric for designing future experiments. This is especially true when working with human subjects and invasive or time consuming data collection methods. In the context of brain-computer interface (BCI) research, maximizing BCI performance is a central goal and so understanding how performance is limited by dataset size or decoding/classification methods is crucial for improving clinical use and understanding the potential role of deep networks in BCIs.

Deep networks are well known for their performance on enormous machine learning datasets. Since neural datasets are typically much smaller, we sought to explore the data efficiency of deep networks in the context of speech classification from CSEPs. We subsampled the training datasets by up to 50 percent in order to estimate accuracy improvements as a function of dataset size. The subsampled training dataset sizes and resulting classification accuracies were then used to estimate the slope of the accuracy as a function of dataset size.

As the fraction of the training set was changed from 0.5 to 1.0, deep network accuracies improve (S3 Fig panel A, solid lines). The accuracy relative to chance is higher for deep networks than for logistic regression (S3 Fig panel A, dotted lines) across dataset fractions for Subjects 1, 3, and 4. S4 Fig contains the same data plotted as raw accuracy and change in accuracy. Deep networks have slopes of Subject 1: 10.1 ± 0.9%, Subject 2: 8.1 ± 1.3%, Subject 3: 2.3 ± 0.3%, and 22.0 ± 2.9% change in accuracy per 1000 training examples (S3 Fig panel B) which are not significantly different from logistic regression slopes. There is no visual indication that performance has saturated for any subject which means the accuracy of classified speech production is in part limited by the amount of training data that can be collected.

### Deep networks have classification confusions that reveal the latent articulatory organization of vSMC

Despite being able to mathematically specify the computations happening everywhere in the model, deep networks are often described as “black boxes”. What deep networks learn and how it depends on the structure of the dataset is not generally understood. This means that deep networks currently have limited value for scientific data analysis because their learned latent structure cannot be mapped back onto the structure of the data. Many current uses of deep networks in scientific applications rely on their high accuracy and do not inspect the network computations [19, 53, 54], although there are results in low dimensional networks [15] and early sensory areas [18]. Nevertheless, deep networks’ ability to consume huge datasets without saturating performance means that expanding their use in science is limited by our understanding of their ability to learn about the structure of data. For the dataset consider in this work, previous studies have shown that an articulatory hierarchy can be derived from the trial-averaged H*γ* amplitude using principal components analysis at hand-selected points in time [20]. Note that the articulatory structure of the consonants and vowels are not contained in the CV labels nor are the individual consonant or individual vowel labels due to the CVs being encoding in a one-hot fashion, i.e., /ba/ (label = 0) is as different from /bi/ (label = 1) as it is from /gu/ (label = 8) according to the CV labels even though they share a consonant (likewise for shared vowels).

To explore whether deep networks can infer this latent structure from the training data, we examined the structure of network output to better understand the organization of deep network syllable representations extracted from vSMC. Deep networks used for classification predict an entire distribution over class labels for each data sample. This learned distribution has been shown to be a useful training target in addition to the thresholded class labels [50, 51]. We clustered these learned representations and compared them to articulatory representations of the CVs.

**Fig 4:**
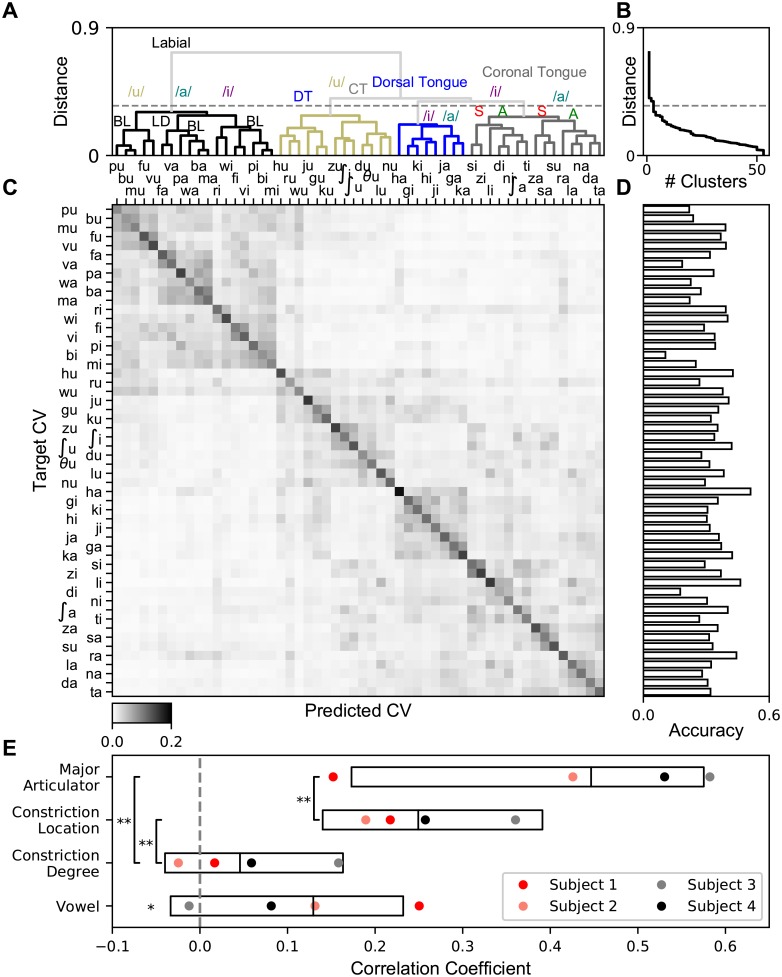
Deep network predictions reveal a latent articulatory hierarchy from single-trial ECoG recordings. **A** The dendrogram from a hierarchical clustering of deep network predictions on the test set averaged across all subjects. The threshold for the colored clusters (dashed gray) is determined from inspection of the number of clusters as a function of distance cutoff shown in **B**. Clusters centroids are labeled with articulatory features shared by leaf CVs. DT: dorsal tongue, CT: coronal tongue, BL: bilabial, LD: labiodental, S: sibilant, A: alveolar. **B** Number of clusters (vertical axis) as a function of the minimum cutoff distance between cluster centroids (horizontal axis). **C** Average predicted probability per CV for Subject 1. CVs are ordered from clustering analysis in **A**. **D** Accuracy of individual CVs. **E** Correlation between pairwise distances in deep network similarity space from **C** compared to distances in an articulatory/phonetic feature space for Major Articulator, Consonant Constriction Location, Consonant Constriction Degree, and Vowel, aggregated across all subjects. Center bar is the median and boundaries are 50% confidence intervals. Colored circles indicate subject medians. **p < 1 × 10^−10^, WSRT, *p < 1 × 10^−4^ t-test, both Bonferroni corrected with *n* = 4.

The dendrogram resulting from agglomerative hierarchical clustering on the trial averaged output of the softmax of the deep network (i.e., before thresholding for classification) averaged across subjects shows clusters spread across scales (Fig 4A). A threshold was chosen by inspection of when the number of clusters as a function of cutoff distance rapidly increased (Fig 4B) and used to color the highest levels of the hierarchy. At the highest level, syllables are confused only within the major articulator involved (lips, back tongue, or front tongue) in the syllable. This is followed by a characterization of the place of articulation within each articulator (bilabial, labio-dental, etc.). At the lowest level there seems to be a clustering across the consonant constriction degree and vowel categories that capture the general shape of the vocal tract in producing the syllable. When ordered by this clustering, the soft confusion matrix (Fig 4C) resulting from the average output of the final layer softmax shows block-diagonal structure corresponding to the articulatory hierarchy. In contrast, deep networks trained on the mel-cepstral coefficients and their time-differences (similar dimensionality to the H*γ* amplitude) show a largely inverted hierarchy, results which mirror those found in more general studies of deep network processing of spoken acoustics [55] (See S5 Fig for this analysis on the data presented here). There is a large amount of variation in the per-CV accuracies (Fig 4D).

This hierarchy can be quantified by comparing the space of deep network prediction probabilities and the space of articulatory features associated with each CV. This comparison was made by correlating pairwise CV distances in these two features spaces across all pairs of CVs. The resulting structure of correlations is consistent with an articulatory organization in vSMC (Fig 4C). The major articulators feature distances are most correlated with the distances between CVs in deep network space, then consonant constriction location, and finally consonant constriction degree and vowel.

Together, these results show that deep networks trained to classify speech from H*γ* activity are learning an articulatory latent structure from the neural data. Qualitatively similar hierarchies can be derived using PCA and logistic regression. Indeed, this structure is in agreement with previous analyses of mean spatial patterns of activity at separate consonant and vowel time points [20] while allowing the consonants and vowels to be part of the same hierarchy. However, the deep network hierarchy has larger correlations and more separation between levels than the hierarchy derived from the Logistic regression model (shown in S6 Fig). Together, these results demonstrate the capacity of deep networks to reveal underlying structure in single-trial neural recordings.

### The high gamma and beta bands show a diversity of correlations across electrodes and CVs

Complex behaviors, such as speech, involve the coordination of multiple articulators on fast timescales. These articulators are controlled by spatially distributed functional areas of cortex. Lower frequency oscillations have been proposed as a coordinating signal in cortex. Previous studies have reported movement- or event-related beta (*β*)-H*γ* desynchronization or decorrelation [33, 34, 42]. The differential structure of these correlations across tasks and functions areas is not commonly analyzed. Since cortex often shows sparse and spatially-differentiated activity across tasks [22], averaging over electrodes and tasks may obscure structure in the cross-frequency relationships.

**Fig 5:**
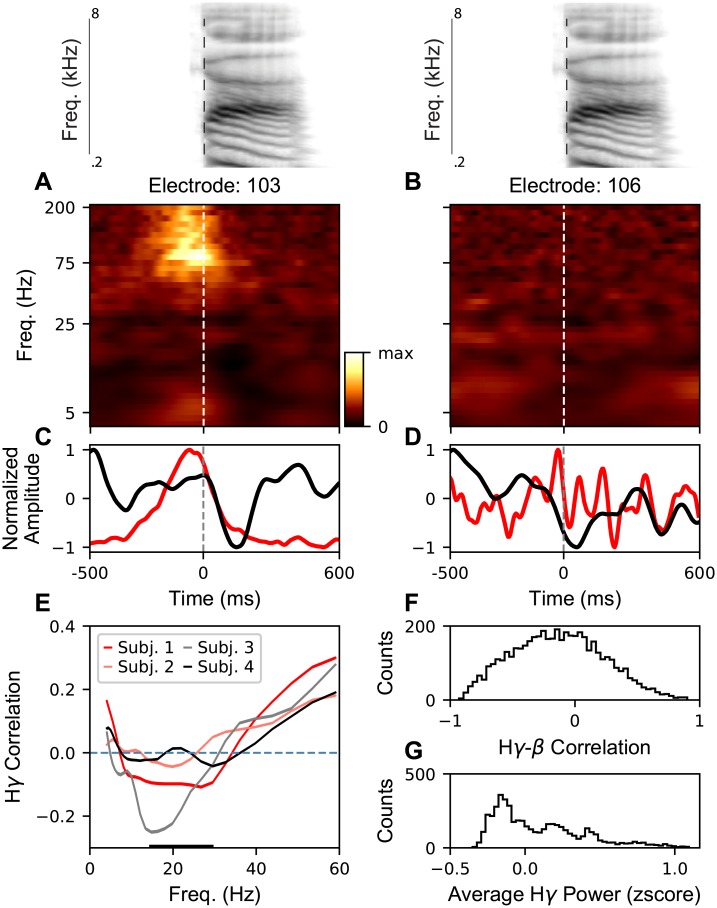
H*γ* and *β* bands show diverse correlation structures across electrodes and CVs. **A**-**B** Average amplitude as a function of frequency and time for an electrode with large activity during /ga/ production and for an electrode with no activity during /ga/ production. **C** and **D** Normalized (-1 to 1) H*γ* (red) and *β* (black) activity from **A** and **B** respectively. Non-trivial temporal relationships can be seen in **C** which are not apparent in **D**. **E** The average correlation (± s.e.m.) between the H*γ* amplitude and the single frequency amplitude is plotted as a function of frequency for each subject. Thickened region of the horizontal axis indicates the *β* frequency range. **F** Histogram of the H*γ*-*β* correlation coefficients for all CVs and electrodes for Subject 1. **G** Histogram of the z-scored H*γ* power near the CV acoustic transition (time = 0) for all CVs and electrodes for Subject 1.

The CV task and grid coverage allow average neural spectrograms (zscored amplitude as a function of frequency and time) to be measured at two electrodes during the production of the syllable \ga\ (Fig 5A and 5B, median acoustic spectrogram is shown above). In order to investigate this, we measured cross frequency amplitude-amplitude coupling (correlation) for individual lower frequency bands and H*γ*. We also examine the aggregate *β* band. Some previous studies attempt to distinguish band-limited and broadband signals in lower frequencies, e.g., *β* [56–58]. However, methods for distinguishing these signals are generally not applied to high sampling rate signals and often require hand-tuning and is an ongoing area of research. As such, here, we are simply looking at correlations between bandpassed signals and not estimating and removing any broadband components. Further modeling would be needed to interpret these signals as correlations between biophysical sources (see Discussion for discussion). Initially, we pool results across all electrodes and CVs in order to replicate methods from previous studies. The H*γ* and *β* amplitudes show a diverse set of temporal relationships in these regions (Fig 5C and 5D). Across frequencies, H*γ* correlation is positive for low frequencies (< 15Hz), then we see negative and near-zero correlations between H*γ* and the *β* range across subjects, and finally the correlation rises for the *γ* range (30–59 Hz) as the frequencies approach H*γ* (Fig 5E). However, these mean correlations mask a broad range of H*γ*-*β* correlations (Fig 5F) across H*γ* activity (across CVs and electrodes). This includes a large number of positive correlations. Similarly, although most of the amplitudes measured are smaller than baseline (Fig 5G), there is a long tail to amplitudes larger than baseline (above 0).

This diversity of correlations and amplitudes across CVs and electrodes indicates there is potentially substructure in the data that is being averaged over. This motivates a disaggregated analysis of the amplitude-amplitude correlations. Naïvely, one might expect to see different cross-frequency relationships in areas that are actively engaged in a task compared to area which are not engaged. The broad coverage of the ECoG grid and the diversity of articulatory movements across the consonants and vowels in the task allow us to investigate whether there is substructure in the amplitude-amplitude cross frequency correlations.

**Fig 6:**
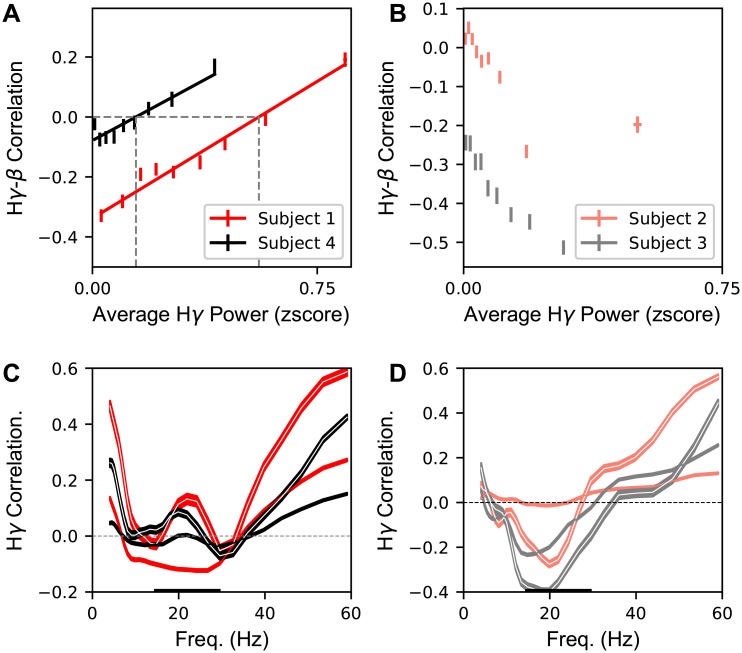
H*γ* and *β* bands show positive correlations at active electrodes which are not found in inactive electrodes for subjects with high classification accuracy. **A** The trial-averaged H*γ*-*β* correlation coefficient across electrodes and CVs is plotted against the average H*γ* power near the CV acoustic transition for Subjects 1 and 4. Solid lines indicate the linear regression fit to the data with positive z-scored amplitude. The vertical dashed gray line indicates the division in average H*γ* power between ‘active’ and ‘inactive’ electrodes for subjects 1 and 4. Data is summarized in nine bins plotted (± s.e.m.) per subject. **B** Same as **A**, but for Subjects 2 and 3, which have a much lower classification accuracy. **C** For the two subjects in **A**, the average (± s.e.m.) correlation is plotted between the H*γ* amplitude and the single frequency amplitude as a function of frequency separately for active (white center line) and inactive (solid color) electrodes. Thickened region of the horizontal axis indicates the *β* frequency range. **D** Same as **C** for subjects in **B**.

In order to investigate this, we grouped the H*γ* activity for each electrode and CV into “active” and “inactive” groups based on the average H*γ* power and computed correlations for these two groups. For the two subjects with high accuracy, we observe a positive correlation between H*γ* power and H*γ*-*β* correlation (Fig 6A). For the two subjects with low CV classification accuracy, we observe a generally negative correlation between H*γ* power and H*γ*-*β* amplitude (Fig 6B).

The H*γ* correlation can be recomputed separately for active and inactive electrodes per CV. For the subjects with high CV classification accuracy (Subjects 1 and 4), we find a novel signature of motor coordination in the active electrodes: a positive correlation in the *β* frequency band (Fig 6C, lines with white centers). This is in contrast to the inactive electrodes, which show small or negative correlation (Fig 6C, solid lines) which is similar to the aggregated results (Fig 5E). For the two subjects with low CV classification accuracy (Subjects 2 and 3), the disaggregated results (Fig 6D) show less dichotomous structure.

Overall, we find that there is structure across bands in addition to cross-frequency relationship with the H*γ* band which has been used in the preceding classification analysis. As far as we are aware, this is the first observation of dichotomous amplitude-amplitude cross frequency correlation during behavior. This observation was only possible because of the broad functional coverage of the ECoG grid and the diverse behaviors represented in the CV task.

### Classification relevant information in lower frequency bands

The gamma and H*γ* band-passed CSEP amplitudes are commonly used both on their own and in conjunction with other frequency bands for decoding produced and perceived speech in humans due to their observed relation to motor and sensory tasks [14, 22, 23, 31, 59, 60]. Other frequency bands have been shown to have amplitude or phase activity which is correlated with H*γ* amplitude or spiking activity [37–40]. Indeed, in the data used in this study, we find amplitude-amplitude correlation structure between H*γ* and lower frequency bands. Although these correlations imply that information is shared between H*γ* and other CSEP frequency bands, it is not known whether the other bands contain additional information about motor tasks beyond H*γ* or whether the information is redundant.

In order to understand the relative information content in CSEP frequency bands, we classified CVs from two different sets of features. Linear classification methods would not give a satisfactory answer to this question since they are limited to simple hyper-plane segmentation of the data which may trivially lead to the result of no information. Indeed, since we have shown that deep networks can outperform linear methods when classifying from H*γ*, they are also candidates for showing whether there is any relevant information in these bands. For the theta, alpha, beta, high beta, and gamma bands, each band’s features were first used for classification and then concatenated with the H*γ* features and used for classification. The raw classification accuracy and improvement beyond H*γ* are two measures that give insight into information content in the other bands.

**Fig 7:**
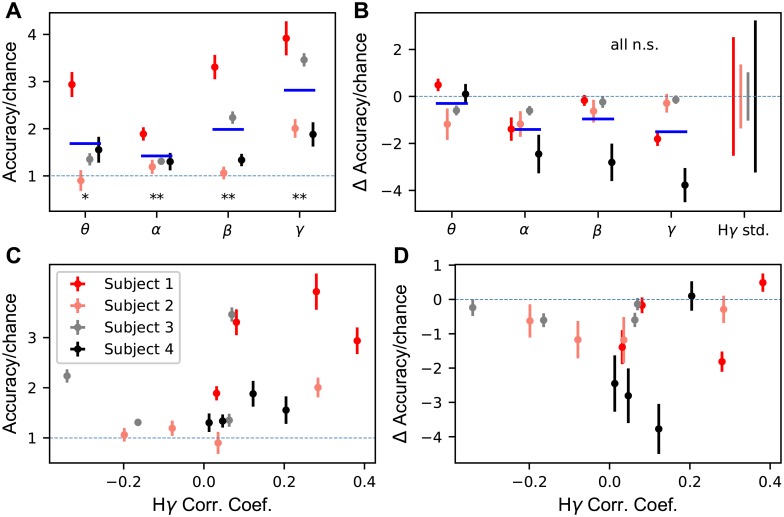
Lower frequency bands do not contribute significant additional information to the CV classification task beyond H*γ*. **A** The average accuracy (± s.e.m., *n* = 10) normalized to chance (chance = 1, dashed blue line) is shown for each frequency band and subject. Subjects are left-right jittered to avoid visual overlap. The solid blue line is the mean across subjects for a single band. **B** Average change in accuracy (± s.e.m., *n* = 10) from H*γ* accuracy normalized to chance when band’s features are concatenated with the H*γ* features. The solid blue line is the mean across subjects for a single band. The H*γ* accuracy cross-validation standard deviation (*n* = 10) normalized to chance is plotted above and below zero in the right-most column for each subject for comparison. **C** Average accuracy (± s.e.m., *n* = 10) normalized to chance (dashed blue line, chance = 1) plotted against the correlation coefficient between H*γ* and the lower frequency band for active electrodes for each band and subject. The blue dashed line indicates chance accuracy. **D** Change in accuracy from H*γ* accuracy normalized to chance plotted against the correlation coefficient between H*γ* and the lower frequency band for active electrodes for each band and subject. The blue dashed line indicates no change in accuracy. **p < 0.001, *p < 0.01, WSRT, n.s., not significant. All Bonferroni corrected with *n* = 5.

Fig 7 shows the accuracies, normalized to chance, across the four subjects. Fig 7A shows the classification accuracies across subjects for single band features. Across subjects, all single bands have CV classification accuracies greater than chance, although subject-to-subject variation is observed. Although this is significantly above chance, the ranges of improvements for the subject means range between 1.5x to 2x chance, a small accuracies compared to H*γ* accuracies which ranged from 6x to 21x chance. For the single band features, accuracy above chance implies that there is relevant information about the task in the bands. Fig 7B shows the chance in classification accuracy relative to H*γ* accuracy, normalized to chance. No bands see a significant improvement in accuracy over the baseline accuracy obtained by classifying from H*γ*. Indeed, all measured mean changes in accuracy are smaller than the cross-validation standard deviations for the H*γ* accuracy. Together, these results show that there is task-relevant information in lower frequency bands, but the information is largely redundant to the information contained in the H*γ* amplitude.

The correlations observed in Figs 5 and 6 imply that there is some shared information between the lower frequency bands and the H*γ* band. However, the classification accuracies from H*γ* alone (Fig 3) are much higher than any other individual frequency band and are not improved by the addition of extra features from lower frequency bands. This shows that the high frequency CSEPs (H*γ* band), which is commonly used in motor decoding, are highly informative signals.

## Discussion

The structure or information content of neural data is often estimated by regressing neural features against known features in the stimulus or behavior. Traditionally, this has been done with linear models, which are often poorly matched to the structure of this relationship. Here, we have shown that deep networks trained on high gamma (H*γ*) cortical surface electrical potentials (CSEPs) can classify produced speech with significantly higher accuracy than traditional linear or single layer models. When classifying syllables, deep networks achieved state-of-the-art accuracy and channel capacity: for the subject with higher accuracy, this was 55.1% and 3.92 bits per syllable. At word durations from Mugler et al. [23] and one CV syllable per word duration, 3.92 bits per syllable corresponds to 7.5 bits per second or 75 words per minute [61]. This could also be combined with a language model to improve accuracy in clinical applications [31] towards the eventual goal of natural spoken speech rates (250-600 words per minute). Generally, we expect that as neuroscientific datasets grow, more modern deep learning techniques and architectures will be used for higher precision such as Residual layers [6], variational auto-encoders [62], and recurrent models for timeseries [7]. Together, these results show that deep networks are a promising analytic platform for brain-computer interface (BCI) for speech prosthetics, an application where high accuracy and high training sample efficiency are crucial. Since deep networks are highly parameterized nonlinear models, their online interactions with learning may be more complex than typical methods [63]. Studying how deep networks behave in an online BCI will be important future step in integrating them into clinical settings.

Training the deep networks described here to high accuracy required an extensive hyperparameter search over model architectures including layer numbers, layer dimensions, and nonlinearity, along with optimization hyperparameters like learning rate, momentum decay, and dropout fraction. In general, the optimal hyperparameters may depend on the latent structure of the dataset being used, however, they may also depend strongly on the size of the dataset in terms of the number of samples or the dimensionality of each sample. Understanding the relationship between the optimal structure of deep networks and the structure of datasets is a future direction of research.

We observed classification accuracies were highest, both relative to chance and linear models, for consonant-vowel syllables compared to the consonants or vowels individually. This is consistent with previous reports on the presence of both anticipatory and perseverative coarticulation effects in vSMC (see also Fig 1E) [21, 23]. Coarticulation refers to the fact that, at a behavioral level, the production of speech phonemes is systematically influenced by the surrounding phonemic context. For communication prosthetics, one might hope to decode the most atomic units, phonemes, and then express the combinatorial complexity of language through combinations of the small number of phonemes. Combined with other studies, the results presented here indicate that coarticulation is a feature of speech motor control that must be accounted for in BCIs.

In contrast to many commercial applications of deep learning, where optimizing prediction accuracy is often the primary goal, in science, it is also desirable to extract latent structure from the data to advance understanding. In the context of the current study, we used deep networks to determine which features of speech production were extracted from the neural activity to solve the classification task. Examination of the consonant-vowel confusions made by the deep networks reveal the underlying articulatory organization of speech production in the vSMC. At the highest level, the deep networks cluster the CVs into the major articulator involved in forming the consonant, i.e. lips, front tongue, or back tongue. The consonant constriction location, e.g. teeth-to-lips versus lips, is in the intermediate level of the hierarchy. Finally, consonant constriction degree and vowel are clustered at the lowest level of the hierarchy. Crucially, the consonant articulatory hierarchy is not present in the CV labels which means that the deep network is extracting this hierarchy from noisy, single-trial CSEPs during training. The articulatory organization we find is consistent with previous studies, which used PCA on the trial-averaged data at specific points in time [20]. However, we note that, while consistent with previous findings, the hierarchy observed here reflects structure across consonants and vowels together. This could not have been examined with the previous methodology, which required analyses at separate time points. In this way, deep networks were able to extract novel, more general structure from the data, and did so with much less human supervision.

As with many studies of human ECoG, there was substantial variability across subjects. Subjects 1 and 4 had the highest CV classification accuracy from H*γ* and also showed similar patterns of H*γ* correlations with lower frequencies (Fig 5) as well as H*γ*-*β* correlation distinctions in active versus inactive electrodes (Fig 6). Subjects 2 and 3 had lower accuracy and had less consistently structured H*γ* correlations. While the precise nature of cross-subject variability is unknown, likely extrinsic contributors are uncontrollable variation in the degree of contact of electrodes with cortex which could impact frequency specific SNR, differences in variance across recording sessions blocks, or degree of subject engagement in task. Further intrinsic sources of variability could include the lack or presence of particular articulator representations in the recorded activity or differing levels of broadband signals in lower frequency bands. However, the frequency specific bump in the *β* range observed in Subjects 1 and 4 is unlikely to be explained by a change in broadband power in active electrodes. This would require the power of the broadband signal to be mainly found in the *β* range and not in the frequencies on either side, which is not consistent with broadband power fluctuations. Interestingly, we found no clear relationship between CV decoding accuracy and the number of trials, suggesting that the variance is due to differences with the underlying signal and not overfitting. Developing machine learning techniques for training networks on CSEPs that generalize across subjects (ECoG grid placement, underlying functional organization, differences in spectral strucure, etc.) is an important direction of future research with broad applications for BCIs [64].

Previous studies of motor cortex have claimed the existence of “beta-desynchronization” (most commonly a decrease in beta amplitude) during motor production [33, 41]. This has led to a variety of hypothesized functions of beta (*β*) band in motor preparation and control, with little consensus across studies. A common methodology in many of these previous studies (especially those done in humans, where the number of samples is small and function of cortex is often sub-sampled) is to aggregate data across all electrodes and tasks. For the two subjects for which there was high-quality decoding accuracy, and thus, likely higher quality CSEP recordings, we found a novel positive coupling, i.e., correlation, between the *β* band and the H*γ* band amplitudes. The positive correlation was band-limited, occurring in the *β* range with a peak near 23Hz, and present at electrode-syllable combinations in which the electrode was active. Thus, uncovering this correlation required that we disaggregate the relation between *β* and H*γ* according to whether an electrode, i.e., articulator, was engaged in the production of a given speech sound. The presence of this coupling is correlated with the classification accuracy from the H*γ* amplitude across subjects. The coupling in engaged functional areas is an example of the possible pitfalls of aggregation across functional areas and specific behaviors or stimuli when the combination of spatial specialization of function and task structure gives rise to sparse activation patterns.

The structure and biophysical origin of broadband and band-limited signals in cortex is an area of active research [35, 56, 57, 59, 65, 66]. In neural power spectra, it has been reported that there are broadband fluctuations in power that can be considered as a separate signal from band-limited signals, e.g., *β* power [56, 57, 67]. Since this signal is broadband, it may mask or enhance cross-frequency correlations between underlying band-limited signals. Several methods have been proposed for estimating broadband signals and separating them from band-limited signals [56–58]. However, these methods are typically applied to ∼1 second windows with a step size that corresponds to ∼1-2 Hz. In this study, the cross-frequency analysis was performed at 200 Hz (although the lowest frequency bands have autocorrelations due to the choice of bandwidths that correspond to ∼4 Hz). At 200 Hz, across the 4 subjects analyzed here, and across all electrodes in vSMC, there are approximately 250 million points at which a broadband signal would need to be extracted. To our knowledge, methods for estimating broadband signals at this scale that are computationally efficient and do not require per-fit hand tuning have not been developed. Developing methods for estimating high sampling rate, continuous broadband signals is an important direction of future research.

Frequency bands besides H*γ* are known to contain information about stimuli, behavioral, and state variables [33, 34, 36, 38, 40, 41, 59]. However, comparisons of task-relevant information across neural signals are rarely made. Information theory provides a way of measuring the amount of information about a task in a neural signal, the mutual information, but measuring mutual information across continuous, high dimensional signals is notoriously difficult. In the context of classifying discrete speech tokens, this information can be approximated through the information transfer rate. Being able to compare information across features is particularly useful for CSEPs which results from a variety of electrical processes in the brain [35]. Since they achieved higher accuracy then linear or single layer methods, deep networks optimized for accuracy can put a tighter bound on the task-relevant information in a set of neural features. We found that, for the amplitudes of frequency bands lower than H*γ*, it is possible to decode speech syllables with above chance accuracy, though at relatively modest levels. Furthermore, when combined with H*γ* features, the relative improvement in accuracy above H*γ* accuracy is small compared to the cross-validation variance. Thus, for BCIs, these results imply that, for the CV task examined here, only H*γ* activity (or higher frequency signals) need be acquired and analyzed: the other parts of the signal may profitably not be acquired to minimize data acquisition hardware and signal-processing in the decoder.

Although deep networks have shown the ability to maximize task performance across scientific and engineering fields, they are still largely black boxes [68]. While there has been some initial investigations [69–73], theoretical and empirical studies have not yet shown how deep networks disentangle the structure of a dataset during training. Currently, deep networks are most commonly used in science in cases where understanding of the deep network’s hidden representation is not needed. While we have taken some initial steps in that direction by examining the networks confusions, revealing how the deep networks disentangled articulatory features from the neural data will be an important extension of this work. An unresoved question is when we can expect deep networks to solve tasks through interpretable latent variables (like phonetic features in the context of speech) and how we can extract these variables from all layers of the learned deep network features (here we only use the learned output probabilities). In general, understanding the interaction between dataset structure and deep network training will make deep networks more broadly useful as a tool for data analytics in science.

Neuroscientists continue to create devices that measure more features in the brain while the stimuli or behavior during data collection become more complex and naturalistic. As the complexity of datasets increase, the tools needed to disentangle and understand these datasets must also evolve. Recently, deep networks have shown promise in analyzing and modeling neural responses in this work and others [17–19]. Moving beyond their utility as high-accuracy regression methods will require a more profound understanding of how deep networks learn to represent complex structure from data sets, and tools to extract that structure so as to provide insights to humans. Indeed, many of the open theoretical and analytical challenges facing deep networks are also core to understanding the brain.

## Supporting information

S1 Table Deep network hyperparameter ranges. Hyperparameters are listed in along with their type and range or options. Nesterov momentum was used as an optimizer for all networks with fixed initial momentum fraction (0.5). The momentum fraction was linearly increased per epoch, starting after the first epoch, to its saturation value. The initial learning rate was exponentially decayed per epoch to a minimum value. Many float hyperparameters were searched in log-space since they typically range over a few orders of magnitude.
(PDF)

S2 Table ANOVA tables. Summary tables for the ANOVA from subsection: Deep networks outperform standard methods for consonant-vowel classification from high gamma amplitude.
(PDF)

S3 Table Classification metric comparison. For each subject and both logistic and deep models, the accuracy, sensitivity, specificity, precision, and F1 score are tabulated for the CV task.
(PDF)

S1 Appendix Optimal deep network hyperparameters for all models, trained model files, and scripts. The optimal hyperparameters for each subject and experiment stored in a YAML file. This also includes a README with links to Docker images which can run the preprocessing code and deep network code, trained model files, and plotting scripts and a link to download the raw data.
(ZIP)

S1 Fig Articulatory features for comparison to deep network prediction features. For each consonant vowel pair (labeled along top and bottom, respectively), a binary feature vector is shown (white indicates the presence of the feature). The grouping into major articulator, consonant constriction location, consonant constriction degree, and vowel features is shown on the right edge.
(TIF)

S2 Fig Classification accuracy of logistic regression versus deep networks for different classification tasks. For **A**-**E**, accuracies (± s.e.m., n = 10) are shown (chance is at the dashed line) independently for each subject and task. Points on the left are multinomial logistic regression accuracy and are connected to the points on the right which are deep network accuracies for each subject. Subject accuracies have been left-right jittered to prevent visual overlap and demarcated with color (legend in **E**). **A-D** Classification accuracy when CV predictions are restricted to consonant constriction location (**A**), consonant constriction degree (**B**), vowel (**C**), or consonant (**D**) classification tasks. **E** Classification of entire consonant-vowel syllables from H*γ* amplitude features.
(TIF)

S3 Fig Classification accuracy improvement normalized to chance as a function of training dataset size for logistic regression versus deep networks. Accuracies (± s.e.m., *n* = 10) are normalized to chance (chance = 1, dashed blue line) independently for each subject. Subject error bars have been left-right jittered to prevent visual overlap and demarcated with color (legend in **A**). **A** Average classification accuracy (± s.e.m., *n* = 10) normalized to chance for the CV task as a function of the fraction of training examples used for logistic regression (dotted lines) and deep networks (solid lines). **B** Change in classification accuracy normalized to chance per 1,000 training examples. The total training set sizes vary significantly between subjects so there is an additional per-subject normalization factor between the slopes in **A** and **B**. p-values were Bonferroni corrected with *n* = 4. n.s., not significant.
(TIF)

S4 Fig Classification accuracy improvement as a function of training dataset size for logistic regression versus deep networks. Accuracies (± s.e.m., *n* = 10) are shown (chance is at the dashed lines) independently for each subject. Subject error bars have been left-right jittered to prevent visual overlap and demarcated with color (legend in **A**). **A** Average classification accuracy (± s.e.m., *n* = 10) for the CV task as a function of the fraction of training examples used for logistic regression (dotted lines) and deep networks (solid lines). **B** Change in classification accuracy per 1,000 training examples. The total training set sizes vary significantly between subjects so there is an additional per-subject normalization factor between the slopes in **A** and **B**.
(TIF)

S5 Fig Deep network predictions reveal a latent acoustic hierarchy from single-trial acoustic recordings. Similar analysis as Fig 5 in the main text for networks trained on mel-cepstral coefficients from Subject 1. **A** The dendrogram from a hierarchical clustering of deep network predictions on the test set from Subject 1. The threshold for the colored clusters (dashed gray) is determined from inspection of the number of clusters as a function of distance cutoff shown in **B**. Clusters centroids are labeled with acoustic features shared by leaf CVs. **B** Number of clusters (vertical axis) as a function of the minimum cutoff distance between cluster centroids (horizontal axis). **C** Average predicted probability per CV for Subject 1. CVs are ordered from clustering analysis in **A**. **D** Accuracy of individual CVs for Subject 1. **E** Correlation between pairwise distances in deep network similarity space from **C** compared to distances in an articulatory/phonetic feature space for Major Articulator, Consonant Constriction Location, Consonant Constriction Degree, and Vowel, aggregated across all subjects. Center bar is the median and boundaries are 50% confidence intervals. Colored circles indicate subject medians.
(TIF)

S6 Fig Logistic regression predictions reveal a latent articulatory hierarchy from single-trial ECoG recordings to a lesser extent. Similar analysis as Fig 5 in the main text for Logistic regression. **A** The dendrogram from a hierarchical clustering of deep network predictions on the test set from Subject 1. The threshold for the colored clusters (dashed gray) is determined from inspection of the number of clusters as a function of distance cutoff shown in **B**. Clusters centroids are labeled with acoustic features shared by leaf CVs. **B** Number of clusters (vertical axis) as a function of the minimum cutoff distance between cluster centroids (horizontal axis). **C** Average predicted probability per CV for Subject 1. CVs are ordered from clustering analysis in **A**. **D** Accuracy of individual CVs for Subject 1. **E** Correlation between pairwise distances in deep network similarity space from **C** compared to distances in an articulatory/phonetic feature space for Major Articulator, Consonant Constriction Location, Consonant Constriction Degree, and Vowel, aggregated across all subjects. Center bar is the median and boundaries are 50% confidence intervals. Colored circles indicate subject medians.
(TIF)
