# Supplementary material for: Deep learning as a tool for neural data analysis: Speech classification and cross-frequency coupling in human sensorimotor cortex
Source: PLoS Comput Biol. 2019 Sep 16;15(9):e1007091. doi: 10.1371/journal.pcbi.1007091 (PMC6762206; doi:10.1371/journal.pcbi.1007091)
Supplement: S1 Table — Hyperparameters are listed in along with their type and range or options. Nesterov momentum was used as an optimizer for all networks with fixed initial momentum fraction (0.5). The momentum fraction was linearly increased per epoch, starting after the first epoch, to its saturation value. The initial learning rate was exponentially decayed per epoch to a minimum value. Many float hyperparameters were searched in log-space since they typically range over a few orders of magnitude. (PDF) [file pcbi.1007091.s001.pdf]

S1 Table: Deep learning as a tool for neural data analysis: speech classification and cross-frequency coupling in human sensorimotor cortex

Jesse A. Livezey, Kristofer E. Bouchard, Edward F. Chang

Fully-connected (FC) deep networks were training using Pylearn2 and Theano [1,2]. Hyperparameters are listed in Table 1. Nesterov momentum was used as an optimizer with fixed initial momentum fraction (0.5). The momentum fraction was linearly increased per epoch, starting after the first epoch, to its saturation value. The initial learning rate was exponentially decayed per epoch to a minimum value. Many float hyperparameters were searched in log-space since they typically range over a few orders of magnitude.

Table 1. Hyperparameters for deep networks

Name	Type	Range/Options
Init. momentum	Float	.5
Terminate after no improvement epochs	Int	10
Num FC Layers	Int	1 : 2
FC dim	Int	dim(task) : 1000
FC layer type	Enum	ReLU, Tanh, Sigmoid
$\log_{10}$ Weight init. scale	Float	-5 : 0
$\log_{10}$ Learning rate init.	Float	-3 : -1
$\log_{10}$ Min. learning rate	Float	-5 : -1
$\log_{10}$ One-minus learning rate decay	Float	-5 : -1
$\log_{10}$ One-minus final momentum	Float	-2 : -3.0102e-1
Momentum saturation epoch	Int	1 : 50
Batch size	Int	15 : 256
Max epochs	Int	10 : 100
One-minus input dropout rate	Float	3.0e-1 : 1
Input dropout rescale	Float	1 : 3
One-minus hidden dropout rate	Float	3.0e-1 : 1
Hidden dropout rescale	Float	1 : 3
$\log_{10}$ L_2 weight decay	Float	-7 : 0
Max filter norm	Float	0 : 3

References

[1] Goodfellow IJ, Warde-Farley D, Lamblin P, Dumoulin V, Mirza M, Pascanu R, et al. Pylearn2: a machine learning research library. arXiv preprint arXiv:13084214. 2013;.

[2] Al-Rfou R, Alain G, Almahairi A, Angermueller C, Bahdanau D, Ballas N, et al. Theano: A Python framework for fast computation of mathematical expressions. arXiv preprint. 2016;.
