# Supplementary material for: Deep learning as a tool for neural data analysis: Speech classification and cross-frequency coupling in human sensorimotor cortex
Source: PLoS Comput Biol. 2019 Sep 16;15(9):e1007091. doi: 10.1371/journal.pcbi.1007091 (PMC6762206; doi:10.1371/journal.pcbi.1007091)
Supplement: S2 Table — Summary tables for the ANOVA from subsection: Deep networks outperform standard methods for consonant-vowel classification from high gamma amplitude. (PDF) [file pcbi.1007091.s002.pdf]

S5 Table: Deep learning as a tool for neural data analysis: speech classification and cross-frequency coupling in human sensorimotor cortex

Jesse A. Livezey, Kristofer E. Bouchard, Edward F. Chang

Table 1. ANOVA Accuracy/chance Results

**Dep. Variable:**	accuracy	**R-squared:**	0.784
**Model:**	OLS	**Adj. R-squared:**	0.779
**Method:**	Least Squares	**F-statistic:**	177.0
**Date:**	Mon, 03 Dec 2018	**Prob (F-statistic):**	6.92e-125
**Time:**	16:24:24	**Log-Likelihood:**	-1047.6
**No. Observations:**	400	**AIC:**	2113.
**Df Residuals:**	391	**BIC:**	2149.
**Df Model:**	8	**Covariance Type:**	nonrobust

	**coef**	**std err**	**t**	**P>|t|**	**[0.025**	**0.975]**
**Intercept**	18.5857	0.605	30.702	0.000	17.396	19.776
**C(subject, 0)[T.1]**	-5.7723	0.475	-12.155	0.000	-6.706	-4.839
**C(subject, 0)[T.2]**	-4.8641	0.475	-10.243	0.000	-5.798	-3.930
**C(subject, 0)[T.3]**	-1.7452	0.475	-3.675	0.000	-2.679	-0.812
**C(complexity, 3)[T.1]**	-13.4608	0.613	-21.956	0.000	-14.666	-12.255
**C(complexity, 3)[T.2]**	-8.6957	0.751	-11.581	0.000	-10.172	-7.219
**C(model, 1)[T.2]**	3.1444	0.751	4.188	0.000	1.668	4.621
**C(complexity, 3)[T.1]:C(model, 1)[T.2]**	-3.0282	0.867	-3.493	0.001	-4.733	-1.324
**C(complexity, 3)[T.2]:C(model, 1)[T.2]**	-2.2729	1.062	-2.140	0.033	-4.361	-0.185

**Omnibus:**	34.883	**Durbin-Watson:**	0.323
**Prob(Omnibus):**	0.000	**Jarque-Bera (JB):**	82.289
**Skew:**	0.442	**Prob(JB):**	1.35e-18
**Kurtosis:**	5.038	**Cond. No.**	13.8
