# Supplementary material for: Deep learning as a tool for neural data analysis: Speech classification and cross-frequency coupling in human sensorimotor cortex
Source: PLoS Comput Biol. 2019 Sep 16;15(9):e1007091. doi: 10.1371/journal.pcbi.1007091 (PMC6762206; doi:10.1371/journal.pcbi.1007091)
Supplement: S3 Table — For each subject and both logistic and deep models, the accuracy, sensitivity, specificity, precision, and F1 score are tabulated for the CV task. (PDF) [file pcbi.1007091.s003.pdf]

S6 Table: Deep learning as a tool for neural data analysis: speech classification and cross-frequency coupling in human sensorimotor cortex

Jesse A. Livezey, Kristofer E. Bouchard, Edward F. Chang

Table 1. Classification metric comparison

**Subject, model**	**Accuracy**	**Sensitivity**	**Specificity**	**Precision**	**F1 score**
Subject 1, deep	55.1 ± 2.3%	54.0 ± 2.3%	99.2 ± 0.0%	56.7 ± 2.7%	68.2 ± 2.4%
Subject 2, deep	15.6 ± 2.2%	14.3 ± 2.7%	98.5 ± 0.0%	15.2 ± 2.8%	20.8 ± 3.3%
Subject 3, deep	21.1 ± 1.6%	20.7 ± 1.8%	98.6 ± 0.0%	20.7 ± 1.8%	31.8 ± 2.5%
Subject 4, deep	43.0 ± 5.3%	43.2 ± 3.7%	99.0 ± 0.1%	47.5 ± 5.1%	56.2 ± 4.8%
Subj. average, deep	33.7 ± 16.4%	33.1 ± 16.4%	98.8 ± 0.3%	35.0 ± 17.8%	44.2 ± 19.2%
Subject 1, logistic	44.6 ± 3.2%	43.8 ± 3.4%	99.0 ± 0.1%	46.1 ± 3.1%	58.5 ± 3.4%
Subject 2, logistic	14.1 ± 2.3%	13.3 ± 2.4%	98.5 ± 0.0%	14.4 ± 2.9%	19.2 ± 3.4%
Subject 3, logistic	18.1 ± 1.5%	17.9 ± 1.6%	98.5 ± 0.0%	18.4 ± 1.9%	28.5 ± 2.6%
Subject 4, logistic	35.3 ± 5.1%	35.8 ± 5.5%	98.8 ± 0.1%	37.6 ± 5.7%	46.4 ± 6.8%
Subj. average, logistic	28.0 ± 12.9%	27.7 ± 13.0%	98.7 ± 0.2%	29.1 ± 13.7%	38.1 ± 15.9%
