# Supplementary figures and images for: Deep learning as a tool for neural data analysis: Speech classification and cross-frequency coupling in human sensorimotor cortex
Source: PLoS Comput Biol. 2019 Sep 16;15(9):e1007091. doi: 10.1371/journal.pcbi.1007091 (PMC6762206; doi:10.1371/journal.pcbi.1007091)

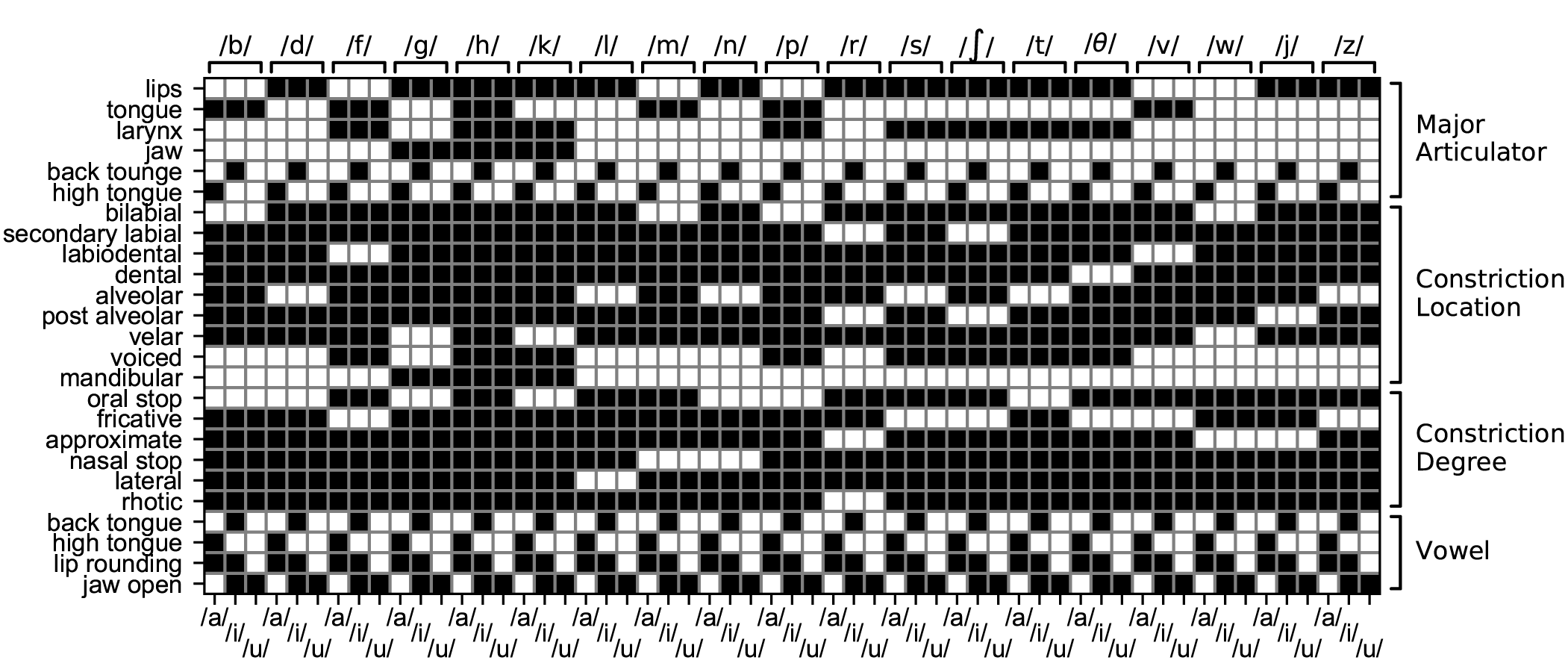

Supplement: S1 Fig — For each consonant vowel pair (labeled along top and bottom, respectively), a binary feature vector is shown (white indicates the presence of the feature). The grouping into major articulator, consonant constriction location, consonant constriction degree, and vowel features is shown on the right edge. (TIF) [file pcbi.1007091.s005.tif]

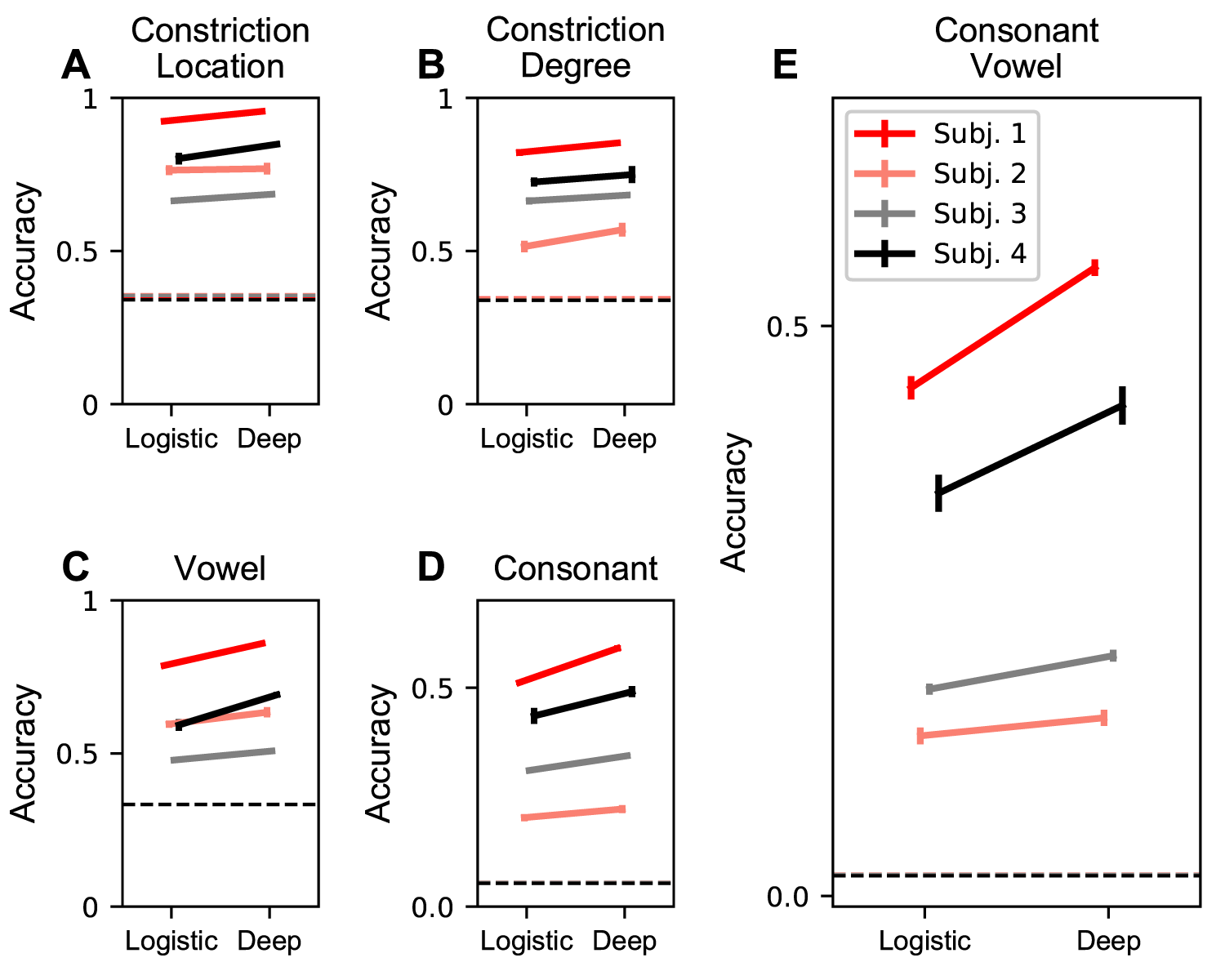

Supplement: S2 Fig — For A-E, accuracies (± s.e.m., n = 10) are shown (chance is at the dashed line) independently for each subject and task. Points on the left are multinomial logistic regression accuracy and are connected to the points on the right which are deep network accuracies for each subject. Subject accuracies have been left-right jittered to prevent visual overlap and demarcated with color (legend in E). A-D Classification accuracy when CV predictions are restricted to consonant constriction location (A), consonant constriction degree (B), vowel (C), or consonant (D) classification tasks. E Classification of entire consonant-vowel syllables from Hγ amplitude features. (TIF) [file pcbi.1007091.s006.tif]

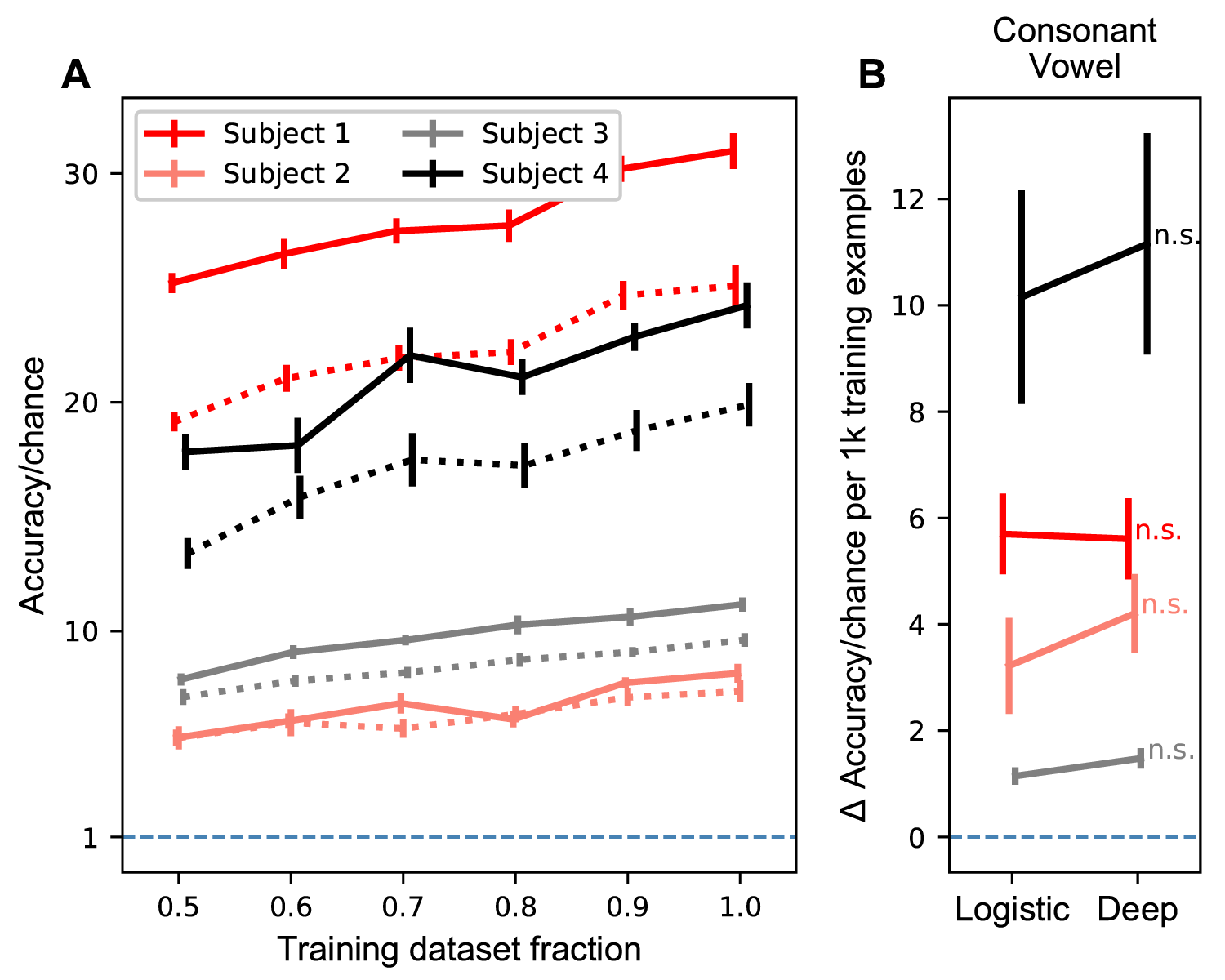

Supplement: S3 Fig — Accuracies (± s.e.m., n = 10) are normalized to chance (chance = 1, dashed blue line) independently for each subject. Subject error bars have been left-right jittered to prevent visual overlap and demarcated with color (legend in A). A Average classification accuracy (± s.e.m., n = 10) normalized to chance for the CV task as a function of the fraction of training examples used for logistic regression (dotted lines) and deep networks (solid lines). B Change in classification accuracy normalized to chance per 1,000 training examples. The total training set sizes vary significantly between subjects so there is an additional per-subject normalization factor between the slopes in A and B. p-values were Bonferroni corrected with n = 4. n.s., not significant. (TIF) [file pcbi.1007091.s007.tif]

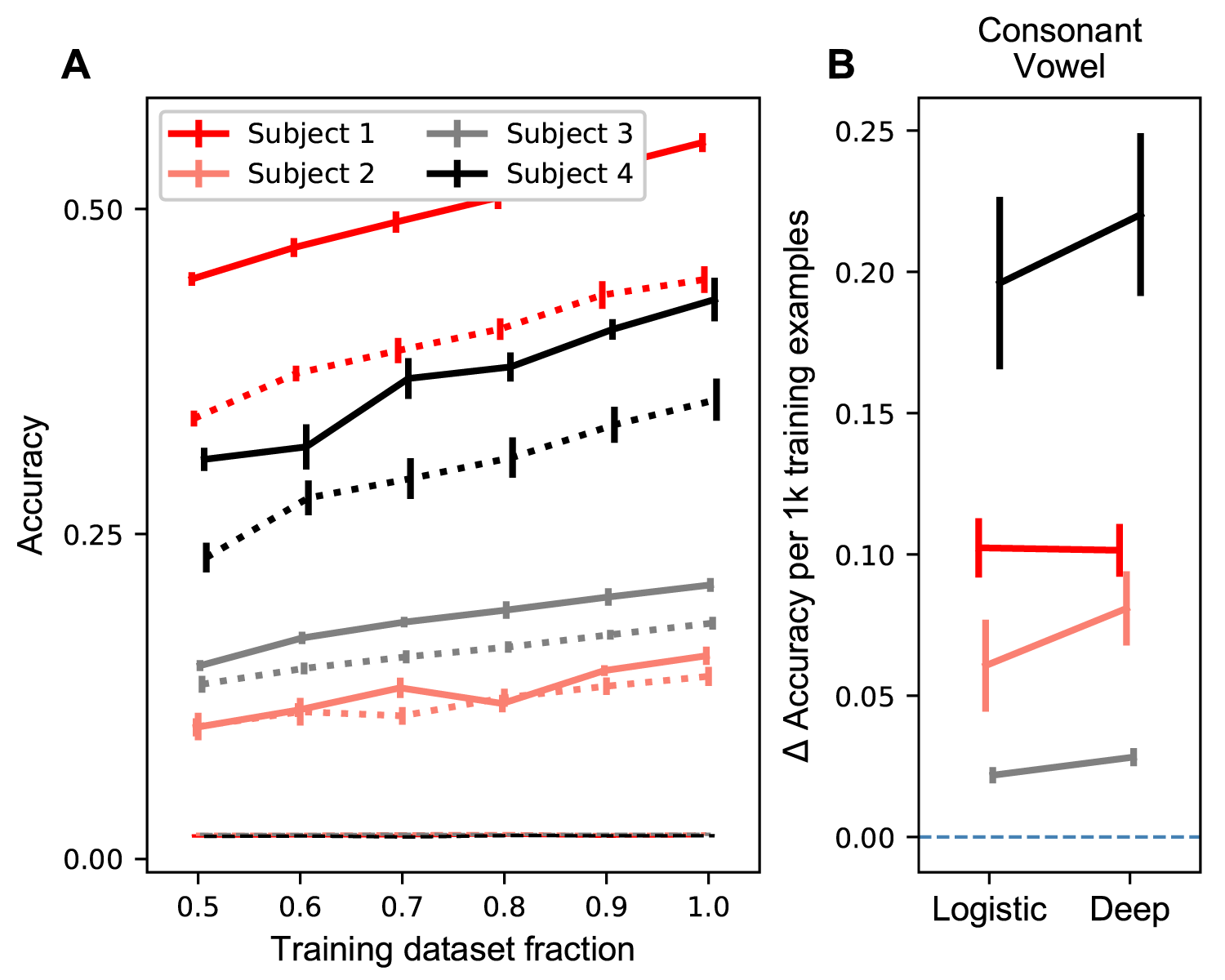

Supplement: S4 Fig — Accuracies (± s.e.m., n = 10) are shown (chance is at the dashed lines) independently for each subject. Subject error bars have been left-right jittered to prevent visual overlap and demarcated with color (legend in A). A Average classification accuracy (± s.e.m., n = 10) for the CV task as a function of the fraction of training examples used for logistic regression (dotted lines) and deep networks (solid lines). B Change in classification accuracy per 1,000 training examples. The total training set sizes vary significantly between subjects so there is an additional per-subject normalization factor between the slopes in A and B. (TIF) [file pcbi.1007091.s008.tif]

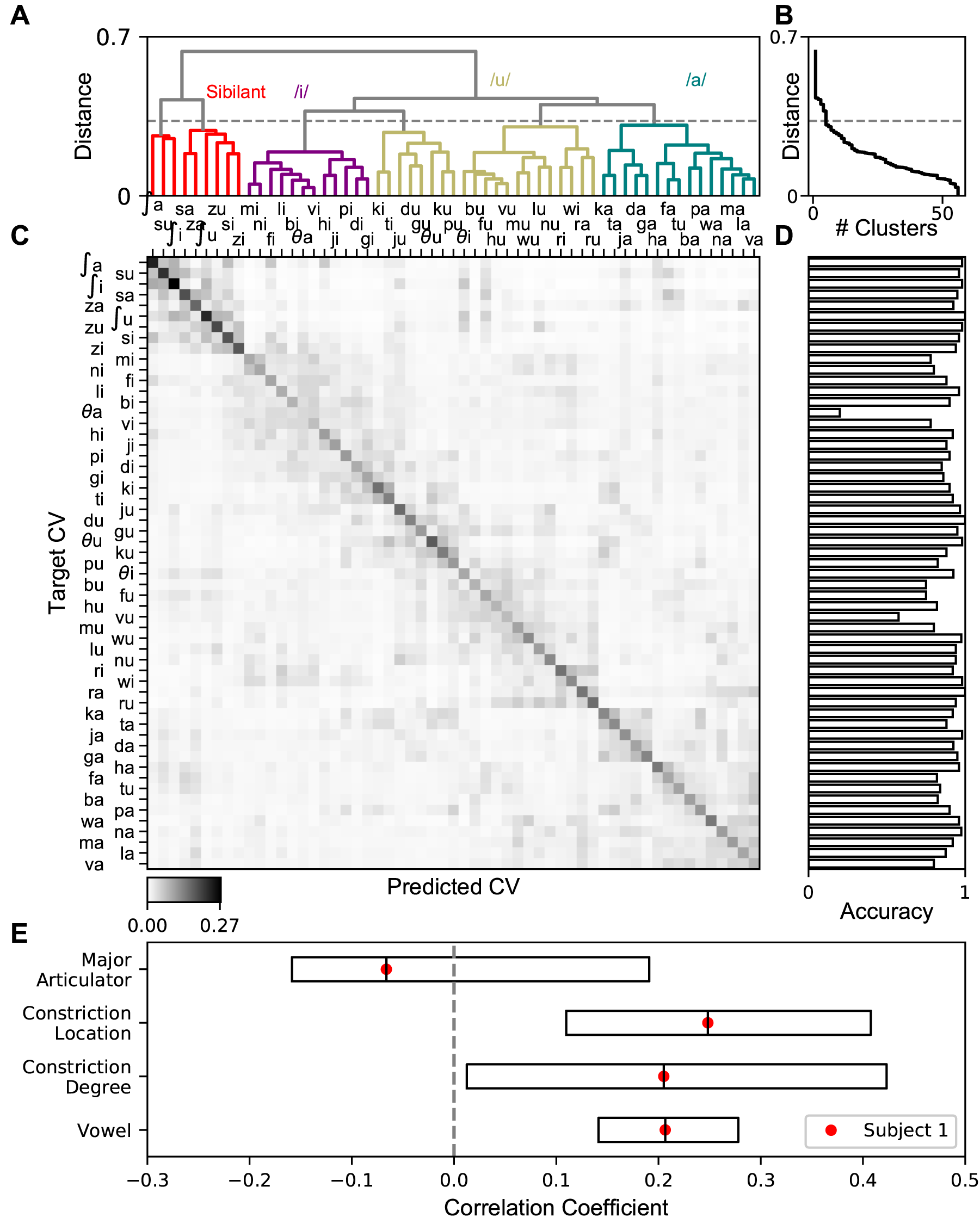

Supplement: S5 Fig — Similar analysis as Fig 5 in the main text for networks trained on mel-cepstral coefficients from Subject 1. A The dendrogram from a hierarchical clustering of deep network predictions on the test set from Subject 1. The threshold for the colored clusters (dashed gray) is determined from inspection of the number of clusters as a function of distance cutoff shown in B. Clusters centroids are labeled with acoustic features shared by leaf CVs. B Number of clusters (vertical axis) as a function of the minimum cutoff distance between cluster centroids (horizontal axis). C Average predicted probability per CV for Subject 1. CVs are ordered from clustering analysis in A. D Accuracy of individual CVs for Subject 1. E Correlation between pairwise distances in deep network similarity space from C compared to distances in an articulatory/phonetic feature space for Major Articulator, Consonant Constriction Location, Consonant Constriction Degree, and Vowel, aggregated across all subjects. Center bar is the median and boundaries are 50% confidence intervals. Colored circles indicate subject medians. (TIF) [file pcbi.1007091.s009.tif]

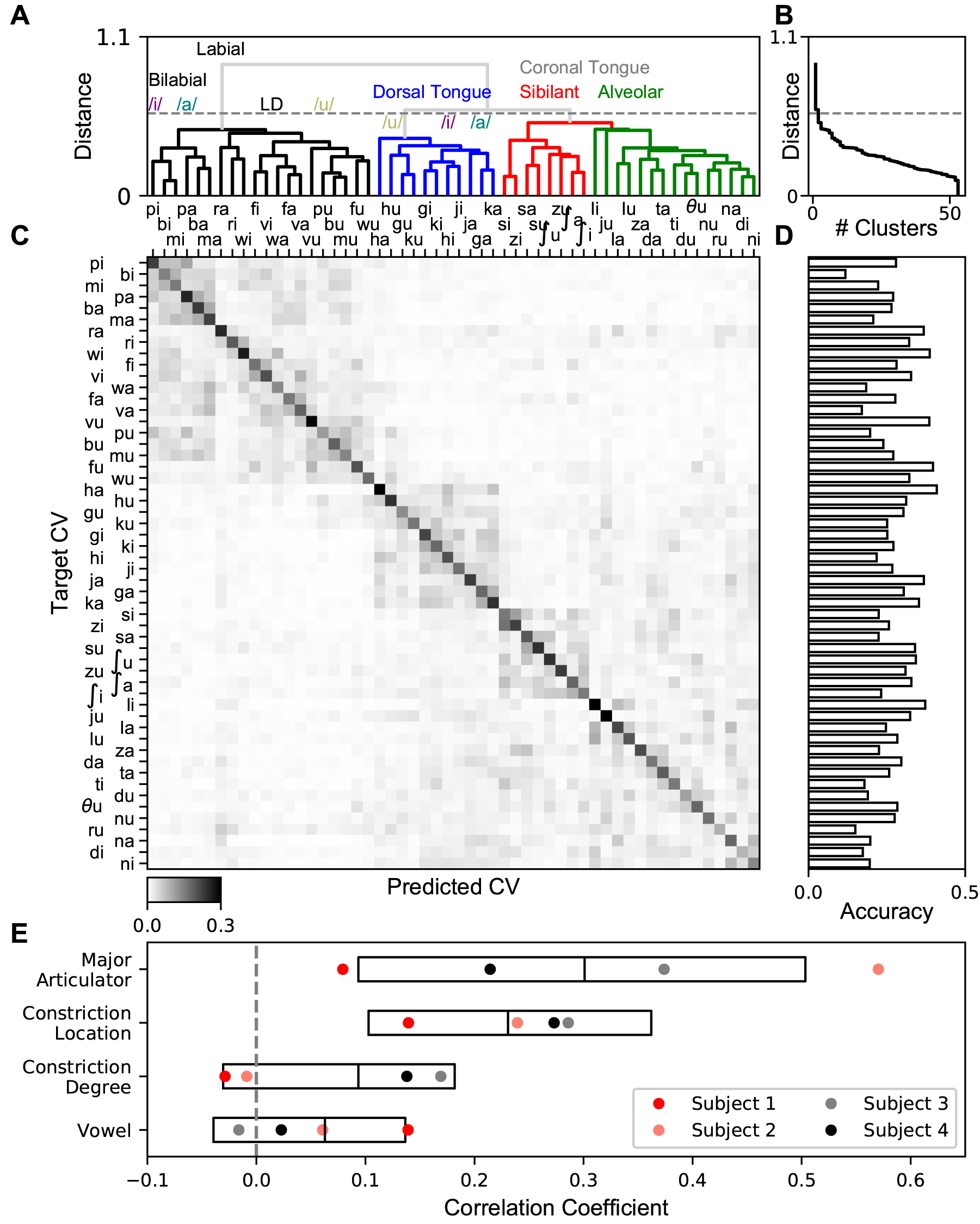

Supplement: S6 Fig — Similar analysis as Fig 5 in the main text for Logistic regression. A The dendrogram from a hierarchical clustering of deep network predictions on the test set from Subject 1. The threshold for the colored clusters (dashed gray) is determined from inspection of the number of clusters as a function of distance cutoff shown in B. Clusters centroids are labeled with acoustic features shared by leaf CVs. B Number of clusters (vertical axis) as a function of the minimum cutoff distance between cluster centroids (horizontal axis). C Average predicted probability per CV for Subject 1. CVs are ordered from clustering analysis in A. D Accuracy of individual CVs for Subject 1. E Correlation between pairwise distances in deep network similarity space from C compared to distances in an articulatory/phonetic feature space for Major Articulator, Consonant Constriction Location, Consonant Constriction Degree, and Vowel, aggregated across all subjects. Center bar is the median and boundaries are 50% confidence intervals. Colored circles indicate subject medians. (TIF) [file pcbi.1007091.s010.tif]
